# Pharmacological mechanisms and clinical impacts of antidiabetic drugs on colorectal cancer risk: a systematic review

**DOI:** 10.3389/fphar.2026.1849647

**Published:** 2026-06-17

**Authors:** Yu Yang, Kan Wang

**Affiliations:** 1 The Second Hospital of Hebei Medical University, Shijiazhuang, Hebei, China; 2 The Fourth Hospital of Hebei Medical University, Shijiazhuang, Hebei, China

**Keywords:** antidiabetic drugs, colorectal cancer, drug repurposing, pharmacology, risk stratification, signaling pathway

## Abstract

Type 2 diabetes mellitus (T2DM) significantly increases colorectal cancer (CRC) risk, driven by shared metabolic and inflammatory pathways. As lifelong glucose-lowering medications are routinely used in T2DM, their pharmacological activities and associated oncological effects have become critical for safety and repurposing. This review systematically summarizes the pharmacological mechanisms, epidemiological evidence, and clinical outcomes of eight antidiabetic drug classes in modulating CRC risk. We highlight drug-specific effects: metformin may act via AMPK/mTOR and immune reprogramming; SGLT-2 inhibitors may exert direct cytotoxicity and indirect metabolic benefits; GLP-1 RAs show class-wide neutrality except high-dose semaglutide; DPP-4 inhibitors display dual pro- and anti-tumor effects; insulin and most secretagogues elevate CRC risk via hyperinsulinemia; while TZDs and AGIs offer modest chemopreventive effects. We conclude that antidiabetic drugs possess pharmacological properties that can inform CRC risk stratification and drug repurposing. Future research should prioritize mechanistic validation and precision pharmacology to translate these findings into clinical practice.

## 1 Introduction

Colorectal cancer (CRC) ranks among the most common malignancies worldwide and is characterized by highly heterogeneous prognosis and treatment responses (Siegel et al., 2023). Accumulating epidemiological evidence indicates that CRC occurs approximately 1.5 times more frequently in patients with type 2 diabetes mellitus (T2DM) than in non-diabetic counterparts, with shared risk factors—including obesity, hyperglycemia, hyperinsulinemia, and chronic inflammation—that contribute to this association (González et al., 2017). Notably, the risk of CRC increases in a clear dose–response manner with longer diabetes duration and higher HbA1c levels—each 1% elevation in HbA1c is associated with a 13% increased risk of CRC (Wang et al., 2024), and progressive worsening of glycemic control is linearly correlated with elevated CRC risk (Mao et al., 2024). This tight biological gradient underscores hyperglycemia and disease chronicity as independent drivers of CRC. Accordingly, a critical challenge in studying antidiabetic drugs is to distinguish drug-specific pharmacological effects from confounding by T2DM severity, glycemic control, and disease duration—an issue explicitly addressed throughout this review.

Over the past decade, the therapeutic landscape for T2DM has expanded considerably, moving beyond conventional agents such as metformin, sulfonylureas, and insulin to include newer drug classes such as sodium-glucose cotransporter-2 (SGLT-2) inhibitors, glucagon-like peptide-1 receptor agonists (GLP-1 RAs), and dipeptidyl peptidase-4 (DPP-4) inhibitors. These agents differ markedly in their mechanisms of action, metabolic effects, and influence on pathways implicated in CRC development. However, inconsistencies in the existing evidence create notable clinical dilemmas. For example, while meta-analyses often report a protective effect of metformin against CRC, bias-adjusted studies challenge this conclusion (Wang and Shi, 2022; Yang et al., 2020; Dickerman et al., 2023; Zhang et al., 2022); DPP-4 inhibitors show contradictory risk signals between randomized controlled trials (RCTs) and observational data (Saito et al., 2021; Fu et al., 2025; Dicembrini et al., 2020; Htoo et al., 2016); and the long-term safety profile of high-dose GLP-1 RAs used for obesity management remains under debate (Hung et al., 2025). Moreover, a persistent translational gap between preclinical findings and human outcomes further complicates the interpretation of available evidence (Koehler et al., 2011; Wenjing et al., 2017).

Despite extensive research, no comprehensive synthesis has integrated evidence across all major antidiabetic drug classes, with a focus on translational mechanisms and personalized clinical decision-making. Most published reviews either concentrate on individual drug classes or omit critical nuances such as analog-specific effects, geographic heterogeneity, and dose-response relationships. This knowledge gap hinders clinicians from optimizing antidiabetic regimens to simultaneously achieve glycemic control and mitigate CRC risk.

To address these limitations, this review systematically evaluates eight major classes of antidiabetic drugs: biguanides (predominantly metformin), SGLT-2 inhibitors, GLP-1 RAs, DPP-4 inhibitors, insulin, thiazolidinediones (TZDs), insulin secretagogues, and alpha-glucosidase inhibitors (AGIs). For each class, we synthesize current evidence on CRC incidence, clinical outcomes (including survival and treatment response), and underlying molecular mechanisms. We also provide integrated clinical recommendations for personalized drug selection in T2DM patients with varying levels of CRC risk and identify priority research directions to resolve remaining uncertainties. To ensure a comprehensive and rigorous synthesis, we conduct a narrative review of systematic reviews, meta-analyses, RCTs, and key observational studies published up to December 2025. Literature was identified through PubMed using search terms combining antidiabetic drug classes and CRC-related outcomes.

## 2 Biguanides and colorectal cancer

### 2.1 Key conclusions

Biguanides (metformin) are classic AMPK-activating antidiabetic agents with pleiotropic pharmacological effects beyond glucose-lowering, including metabolic reprogramming, immune modulation, and tumor-suppressive activities in preclinical models (Huang XW. et al., 2023; Daniłowska et al., 2024). Phosphoproteomic analyses further reveal that metformin extensively remodels cell signaling networks, albeit with marked heterogeneity across CRC cell lines (Salovska et al., 2023).

The epidemiological evidence presents a complex picture: meta-analyses of observational studies report inverse associations between metformin use and CRC incidence, whereas bias-adjusted studies indicate neutral effects, suggesting correlation rather than causality (Wang and Shi, 2022; Yang et al., 2020; Dickerman et al., 2023; Zhang et al., 2022; Chen et al., 2023; Rangraze et al., 2025). Beyond primary prevention, metformin use is associated with improved survival outcomes in T2DM patients with established CRC (Feng and Qin, 2021; Christou et al., 2022; Tarhini et al., 2022).

Despite these observational associations and a strong mechanistic rationale (detailed in Section 2.3), definitive proof of causality from large-scale RCTs remains lacking, and significant heterogeneity exists across patient subgroups and study designs.

### 2.2 Epidemiological and clinical outcome evidence

#### 2.2.1 Evidence for protective association with CRC incidence

Multiple meta-analyses of observational studies support an inverse association between metformin use and reduced CRC incidence (Wang and Shi, 2022; Yang et al., 2020; Chen et al., 2023; Rangraze et al., 2025). A meta-analysis of 17 observational studies involving over 1 million T2DM patients found metformin use was associated with a 12% reduced risk of CRC (adjusted RR = 0.88, 95% CI: 0.83–0.94) (Yang et al., 2020). Another comprehensive meta-analysis of 65 studies reported an associated 38% reduction in CRC risk (RR = 0.62, 95% CI: 0.51–0.76) (Rangraze et al., 2025). This association appears consistent across American and European populations and extends to high-risk groups, such as patients with both inflammatory bowel disease and T2DM, where metformin use was associated with a 56% lower CRC risk (adjusted HR = 0.44, 95% CI: 0.29–0.68) (Huang et al., 2025). Mendelian randomization analysis further supports biological plausibility for a chemopreventive effect, showing metformin use is associated with downregulation of cancer field effect genes in healthy colon mucosa (Moratalla-Navarro et al., 2024). Notably, all these findings represent observational associations and do not confirm a causal effect of metformin on CRC incidence.

#### 2.2.2 Conflicting evidence and methodological considerations

However, several well-designed studies that rigorously avoid immortal time bias and residual confounding challenge the causal interpretation of the observed protective association and demonstrate neutral effects of metformin on CRC incidence (Dickerman et al., 2023; Zhang et al., 2022). A cohort study of over 41,000 newly diagnosed T2DM patients using time-dependent Cox regression found no significant association between metformin use and CRC incidence (HR = 0.88, 95% CI: 0.68–1.13) (Zhang et al., 2022). Similarly, a target trial emulation using UK electronic health records concluded that metformin therapy did not meaningfully influence CRC incidence among T2DM patients or the general population (Dickerman et al., 2023). Furthermore, adding low-dose aspirin to metformin therapy in T2DM patients did not confer an additional reduction in CRC risk, suggesting that the observed benefit of metformin may not be easily augmented by common chemopreventive agents (Shami et al., 2023). These discrepancies highlight that observational estimates are strongly affected by study design and bias, and that prior findings reflect correlation rather than proven causality. The divergent results between conventional meta-analyses (reporting protective associations) and bias-adjusted studies (reporting neutral effects) can be primarily attributed to immortal time bias. In typical observational analyses, the time between diabetes diagnosis and metformin initiation is often misclassified as “exposed” time, creating a spurious survival advantage for metformin users. Additionally, confounding by indication may play a role: healthier, more adherent patients are more likely to be prescribed metformin, whereas those with more severe diabetes or comorbidities are more likely to receive insulin or sulfonylureas. When these biases are addressed through time-dependent Cox regression or target trial emulation, the neutral findings suggest that metformin does not causally reduce CRC incidence (Dickerman et al., 2023; Zhang et al., 2022).

#### 2.2.3 Improved survival in patients with established CRC

Compared with the conflicting data on CRC incidence, the evidence for associations between metformin use and improved clinical outcomes in T2DM patients with established CRC is more consistent (Feng and Qin, 2021; Christou et al., 2022; Tarhini et al., 2022). A retrospective cohort study found metformin users had significantly higher rates of 2-year overall survival (OS) (86.9% vs. 71.0%) and better disease-free survival (DFS) (Tarhini et al., 2022). A nationwide data linkage study showed that each 10% increase in 1-year adherence to metformin was associated with reduced cancer-specific mortality in CRC patients (adjusted HR = 0.94, 95% CI: 0.91–0.96) (Feng and Qin, 2021). A pooled analysis of three adjuvant trials in stage II–III colon cancer patients found that the detrimental effects of diabetes on time to recurrence and OS were attenuated in metformin users (Christou et al., 2022). In metastatic CRC, metformin use has been associated with improved progression-free survival (PFS) (14.0 vs. 9.9 months) (Erdat et al., 2025). These findings represent robust observational associations and not definitive causal evidence of metformin improving CRC survival.

#### 2.2.4 Synergy with conventional therapy and subgroup variability

Metformin has been associated with enhanced efficacy of standard CRC treatments (Wang et al., 2025; Bragagnoli et al., 2021; Anselmino et al., 2021; Lai et al., 2023). It can reverse 5-fluorouracil (5-FU) resistance induced by radiotherapy by modulating folate metabolism and exerts synergistic cytotoxic effects with 5-FU and irinotecan in refractory CRC (Wang et al., 2025; Bragagnoli et al., 2021). Additionally, metformin combined with propranolol has shown efficacy in preventing 5-FU resistance in preclinical models (Anselmino et al., 2021). In rectal cancer patients receiving neoadjuvant chemoradiotherapy, metformin use was associated with significantly higher rates of tumor regression grade and T downstaging (Lai et al., 2023). However, potential benefits appear heterogeneous across subgroups. For example, a Finnish cohort study found only a weak association between pre-diagnostic metformin use and reduced CRC-related death in colon cancer, with no significant effect for post-diagnostic use or in rectal cancer (Erkinantti et al., 2022a; Erkinantti et al., 2022b). Visceral obesity may modify the effect, with metformin associated with improved recurrence-free survival primarily in early-stage CRC patients with a high visceral fat index (Vedire et al., 2023).

Importantly, not all combination strategies have proven successful. A phase II trial of nivolumab combined with metformin in patients with treatment-refractory microsatellite stable (MSS) metastatic CRC showed no objective responses, despite some observed immunomodulatory effects in tumor tissue (Akc et al., 2023). This underscores the challenge of translating promising preclinical findings of synergy between metformin and immunotherapy into clinical benefit for advanced, refractory CRC.

### 2.3 Pharmacological mechanisms

The preclinical mechanistic pathways that may underlie the observed observational associations of metformin with CRC outcomes are pleiotropic, involving metabolic reprogramming, immune modulation, gut microbiome changes, oncogenic signaling inhibition, and suppression of cancer stemness and metastasis. These findings support biological plausibility but do not confirm clinical causality in humans.

#### 2.3.1 Metabolic reprogramming

Metformin substantially alters CRC cell metabolism. It reprograms tryptophan metabolism by downregulating MYC and the transporter SLC7A5 in CRC cells, thereby restoring tryptophan availability for CD8^+^ T cells and enhancing their antitumor function (Huang XW. et al., 2023). Nuclear VCP promotes colorectal cancer progression by upregulating fatty acid oxidation (FAO) via the VCP-HDAC1-CPT1A axis, and a VCP inhibitor can block FAO upregulation induced by metformin (Huang YW. et al., 2023). Furthermore, metformin regulates folate metabolism to reverse chemotherapy resistance and inhibits the urea cycle, reducing putrescine generation through AMPK/p53 pathway activation (Wang et al., 2025; Zhang T. et al., 2021).

#### 2.3.2 Modulation of the tumor microenvironment and immune response

Metformin reshapes the immunosuppressive tumor microenvironment in CRC. In CRC patients with T2DM, metformin use is associated with increased densities of CD3^+^ and CD8^+^ tumor-infiltrating lymphocytes and tertiary lymphoid structures (Saito et al., 2020). It promotes the polarization of tumor-associated macrophages (TAMs) towards the anti-tumor M1 phenotype by inhibiting HIF-1α and mTOR signaling and suppresses pro-inflammatory signaling pathways (e.g., NF-κB/NLRP3/IL-1β axis) in immune cells exposed to tumor signals (Cao et al., 2023; Liu et al., 2025; Djebri et al., 2025). In colitis-associated CRC models, metformin suppresses tumorigenesis by inhibiting the TLR4/MyD88/NF-κB/MAPK pathway (Lai et al., 2025).

#### 2.3.3 Gut microbiome modulation

The gut microbiome is a key mediator. In obese mice, oral metformin suppresses colon tumor growth by increasing the abundance of short-chain fatty acid-producing microbes (Broadfield et al., 2022). It also attenuates *Fusobacterium* nucleatum-induced tumorigenesis in susceptible mice by modulating the gut microbial composition (Huang et al., 2020).

#### 2.3.4 Inhibition of oncogenic signaling and induction of cell death

Metformin activates AMPK, leading to inhibition of mTORC1 signaling and subsequent suppression of cell proliferation (Daniłowska et al., 2024; Sun et al., 2023). Beyond this canonical pathway, large-scale phosphoproteomic analyses have revealed that metformin’s primary effect is to extensively remodel cell signaling networks, rather than alter total protein expression, and that this signaling response is highly heterogeneous across molecularly diverse CRC cell lines (Salovska et al., 2023). This heterogeneity may underlie differential clinical responses. It downregulates inhibin beta A (INHBA) to suppress the TGF-β/PI3K/AKT pathway and inhibits the NLRP3 inflammasome (Liu et al., 2025; Xiao et al., 2022). Metformin induces cell cycle arrest, apoptosis, and ferroptosis. For instance, it sensitizes TRAIL-resistant CRC cells to apoptosis by blocking the AKT/NF-κB pathway (Lee et al., 2025). The triple combination of metformin, thymoquinone, and 5-FU demonstrates superior pro-apoptotic and anti-proliferative effects in CRC cells compared to dual therapies or monotherapies, which is mediated through stronger suppression of the PI3K/mTOR/HIF1α pathway and augmentation of oxidative stress (Farrash et al., 2023). Additionally, in combination with curcumin, metformin enhances ferroptosis by increasing reactive oxygen species (ROS) and downregulating xCT (Chuang et al., 2025).

#### 2.3.5 Targeting cancer stem cells and metastasis

Metformin also targets cancer stemness and metastasis in CRC. It upregulates miR-342-3p in CD133+ colon cancer stem cells, thereby reducing CD133 expression, a key marker associated with stem cell properties (Zhang YQ. et al., 2021). Functional screens have identified several microRNAs that sensitize CRC cells to metformin’s anti-proliferative effects by modulating metabolism, apoptosis, and cell cycle (Orang et al., 2022). Furthermore, metformin upregulates hsa-miR-1306-3p to suppress the expression of Four-jointed box kinase 1 (FJX1), thereby contributing to reduced viability (Kim et al., 2025). It also suppresses epithelial-mesenchymal transition (EMT) by promoting the redistribution of E-cadherin and β-catenin to cell-cell junctions and downregulating focal adhesion kinase (FAK) (Sugiura et al., 2022; Amable et al., 2020).

### 2.4 Clinical implications and future directions

Before presenting specific clinical implications, it is important to clarify the evidentiary basis of the recommendations that follow. Unless otherwise specified, the suggestions in this section are derived from observational studies and preclinical mechanistic data, which provide correlational rather than causal evidence. Readers should therefore interpret these recommendations as exploratory and hypothesis-generating rather than definitive clinical guidelines. Definitive causal inference and strong clinical recommendations await confirmation from large-scale, long-term randomized controlled trials.

#### 2.4.1 Implications for clinical practice

##### 2.4.1.1 Risk-benefit in T2DM management

Given the consistent observational inverse association for CRC risk and robust associated survival benefits, metformin may be considered a first-line agent for T2DM patients, particularly those at elevated CRC risk (e.g., family history, personal history of polyps, obesity) (Wang and Shi, 2022; Yang et al., 2020; Chen et al., 2023; Rangraze et al., 2025; Feng and Qin, 2021; Christou et al., 2022; Tarhini et al., 2022). These recommendations are based on observational associations and mechanistic plausibility, not definitive causal evidence.

##### 2.4.1.2 Adjuvant and combination potential

Preclinical and clinical observational evidence supports associations between metformin use and enhanced efficacy of chemotherapy and radiotherapy, warranting exploration of metformin as an adjuvant therapy in CRC treatment regimens, especially in diabetic patients (Wang et al., 2025; Bragagnoli et al., 2021; Anselmino et al., 2021; Lai et al., 2023). However, clinicians should be aware that combination with immune checkpoint inhibitors has not yet demonstrated efficacy in refractory settings (Akc et al., 2023).

##### 2.4.1.3 Awareness of limitations and heterogeneity

Clinicians should be aware that the apparent magnitude of protective effect may be overstated in much of the observational literature due to methodological limitations and biases. The benefit may also be heterogeneous across cancer subtypes (colon vs. rectal) and patient characteristics (e.g., visceral obesity, tumor molecular profile).

#### 2.4.2 Limitations of current evidence

##### 2.4.2.1 Predominance of observational data

The evidence base is predominantly observational and prone to immortal time bias, confounding by indication, and residual confounding (Dickerman et al., 2023; Zhang et al., 2022).

##### 2.4.2.2 Inconsistent clinical trial results

While some clinical trials show promise, others, such as a phase II trial combining metformin with nivolumab in microsatellite stable (MSS) metastatic CRC, showed no evidence of efficacy despite observed immunomodulatory effects (Akc et al., 2023).

##### 2.4.2.3 Translational gaps and model limitations

Preclinical studies often use supraphysiological drug concentrations, and the relevance of animal models to human disease is limited. Advanced models, such as patient-derived organoids (PDOs), are crucial for bridging this gap, as demonstrated by high-throughput drug screening in a biobank of high-risk colorectal adenoma organoids, which validated metformin as a potential chemopreventive agent (Luo et al., 2023). However, even sophisticated 3D models like CRC tumoroids show complex and dose-dependent responses to metformin, involving modulation of shared autophagy/apoptosis pathways, highlighting the difficulty in predicting clinical efficacy from *in vitro* models (Shabkhizan et al., 2025).

##### 2.4.2.4 Unclear optimal use and formulation

The optimal dose, treatment duration, and patient subgroups most likely to benefit remain undefined. Research into novel formulations, such as mitochondria-modulating liposomes co-loaded with metformin and 2-deoxy-D-glucose (2-DG), shows promise in reversing radioresistance in preclinical models, but this represents a significant departure from standard metformin therapy and is in early stages of development (Li JM. et al., 2024).

#### 2.4.3 Future research priorities

##### 2.4.3.1 Definitive RCTs

Large-scale, long-term RCTs are urgently needed to establish causal evidence for both primary CRC prevention and adjuvant therapy. Trials should stratify patients by T2DM status, metabolic phenotypes (e.g., visceral obesity), and tumor molecular subtypes.

##### 2.4.3.2 Precision medicine and biomarker discovery

Research should focus on identifying predictive biomarkers (e.g., tumor AMPK/mTOR pathway status, immune microenvironment profile, gut microbiome signatures) to guide patient selection. Insights from phosphoproteomic studies on intracellular signaling heterogeneity and microRNA regulators should be harnessed for this purpose (Salovska et al., 2023; Orang et al., 2022; Kim et al., 2025).

##### 2.4.3.3 Mechanistic and combination therapy research

Further elucidation of mechanisms, especially the crosstalk between the gut microbiome and immune modulation, is needed. Studies should explore synergistic combinations with chemotherapy, radiotherapy, immunotherapy, and targeted agents, building on promising yet complex preclinical findings, such as triple-drug synergies (Farrash et al., 2023).

##### 2.4.3.4 Leveraging advanced models

The use of clinically relevant models, such as PDOs and 3D tumoroids, should be prioritized to improve the translatability of preclinical findings and refine metformin-specific dose-response relationships before clinical testing (Luo et al., 2023; Shabkhizan et al., 2025).

## 3 SGLT-2 inhibitors and CRC

### 3.1 Key conclusions

SGLT-2 inhibitors are antidiabetic agents that inhibit renal glucose reabsorption and have demonstrated activity in both preclinical and clinical settings. Observational studies consistently report an inverse association between SGLT-2 inhibitor use and lower CRC incidence, compared with DPP-4 inhibitors (Suzuki et al., 2024; Sung et al., 2024; Hajishah et al., 2025). However, a two-sample Mendelian randomization study found no causal evidence supporting an effect of SGLT-2 inhibitors on CRC risk (Ju et al., 2024). SGLT-2 inhibitor use is associated with improved overall survival and progression-free survival in T2DM patients with established CRC (Chiang et al., 2024). Mechanistically, some SGLT-2 inhibitors exert direct cytotoxicity via the SGLT2/SIRT3 axis in preclinical models (Anastasio et al., 2024), whereas others lack direct anti-proliferative effects and act only indirectly through metabolic and anti-inflammatory improvements (Kato et al., 2019). Collectively, current evidence supports observational associations and biological plausibility, but not definitive causal proof for CRC risk reduction.

### 3.2 Epidemiological and clinical outcome evidence

#### 3.2.1 Consistent protective association with CRC incidence

Multiple large-scale real-world observational studies demonstrate a consistent inverse association between SGLT-2 inhibitor use and lower CRC incidence in T2DM patients (Suzuki et al., 2024; Sung et al., 2024; Hajishah et al., 2025). A propensity score-matched nationwide study found SGLT-2 inhibitors were associated with a 29% lower CRC risk compared with DPP-4 inhibitors (Suzuki et al., 2024). Another real-world study confirmed that dapagliflozin and empagliflozin were associated with significantly lower CRC risk relative to DPP-4 inhibitors (Sung et al., 2024). A meta-analysis of 17 cohort studies further supported a trend toward reduced CRC risk with SGLT-2 inhibitors (pooled RR = 0.80) (Hajishah et al., 2025). All these findings represent associations, not proven causality.

#### 3.2.2 Potential broad-spectrum anticancer effects

The observed inverse association of SGLT-2 inhibitors appears to extend beyond CRC. The meta-analysis indicated that SGLT-2 inhibitor use was associated with significantly lower risks of liver (RR = 0.76), lung (RR = 0.87), and prostate (RR = 0.75) cancers compared to DPP-4 inhibitor use, suggesting a broad correlational signal rather than organ-specific effects (Hajishah et al., 2025). These findings remain associative and do not confirm causal anticancer effects.

#### 3.2.3 Improved survival in patients with established CRC

Beyond primary prevention, SGLT-2 inhibitor use is associated with substantially better survival outcomes in T2DM patients with established CRC. A propensity-matched retrospective study showed that SGLT-2 inhibitor users had higher 5-year OS and PFS rates, with a 50%–70% reduction in mortality and progression risk (Chiang et al., 2024). These results represent robust observational associations; causal evidence is lacking.

#### 3.2.4 Methodological limitations and causal inference challenges

Notably, a two-sample Mendelian randomization study found no causal evidence linking SGLT-2 inhibitors to reduced CRC risk (Ju et al., 2024). This discrepancy highlights that observational associations do not equate to causality, and residual confounding or metabolic differences may drive the observed benefits (Suzuki et al., 2024; Sung et al., 2024; Hajishah et al., 2025; Ju et al., 2024). Definitive RCTs are required to confirm causal effects on CRC incidence and survival.

Several factors may explain the discrepancy between observational studies (reporting protective associations) and the Mendelian randomization study (finding no causal evidence). First, MR studies use genetic variants as proxies for drug exposure and cannot capture dynamic factors such as drug dosage, treatment duration, adherence, or drug-specific effects—all of which may be critical for CRC risk modulation. Second, different SGLT-2 inhibitors (e.g., canagliflozin vs. tofogliflozin) have distinct mechanisms of action, with some exerting direct cytotoxicity and others acting indirectly; MR analyses aggregate across drug classes and may miss these agent-specific differences (Anastasio et al., 2024; Kato et al., 2019). Third, most observational studies had relatively short follow-up durations (Suzuki et al., 2024), whereas CRC carcinogenesis typically requires longer latency periods; thus, the observed associations may reflect short-term metabolic improvements rather than true chemoprevention.

Variations across observational studies themselves may also contribute to heterogeneity. Differences in baseline CRC risk across populations, choice of comparator drugs (DPP-4 inhibitors vs. other antidiabetic agents), and specific SGLT-2 inhibitor analogs studied can all influence effect estimates (Hajishah et al., 2025). Therefore, the neutral MR finding does not necessarily refute the observational associations but rather highlights the need for long-term RCTs that account for drug-specific and dose-dependent effects.

### 3.3 Pharmacological mechanisms

The preclinical mechanisms underlying the observed observational associations of SGLT-2 inhibitors with CRC outcomes include both indirect metabolic modulation and agent-specific direct cytotoxicity (Anastasio et al., 2024; Kato et al., 2019). These pathways support biological plausibility but do not confirm clinical causality in humans.

#### 3.3.1 Indirect pathway: metabolic and microenvironmental modulation

*In vivo* studies using diabetic and obese animal models demonstrate that SGLT-2 inhibitors suppress CRC tumorigenesis primarily through systemic metabolic regulation and anti-inflammatory effects. Specifically, in azoxymethane-induced diabetic db/db mice, treatment with the SGLT-2 inhibitor tofogliflozin significantly suppressed the development of colorectal neoplastic lesions and β-catenin-accumulating crypts (Kato et al., 2019). This effect was associated with improved glycemic control, reduced serum TNF-α levels, downregulated pro-inflammatory gene expression in white adipose tissue, and reduced macrophage infiltration (Kato et al., 2019). Notably, tofogliflozin showed no direct anti-proliferative effects on SGLT-2-expressing human CRC cells *in vitro* (Kato et al., 2019). This supports the importance of indirect, host-mediated mechanisms (e.g., glycemic control, anti-inflammation) in its *in vivo* anticancer activity, rather than direct cellular targeting.

#### 3.3.2 Direct pathway: cellular metabolic stress and death induction

In contrast, certain SGLT-2 inhibitors exert direct cytotoxic effects on CRC cells through distinct molecular pathways *in vitro*. Specifically, treatment with canagliflozin (50 µM for 72 h) induced G1-phase cell cycle arrest (*P* < 0.001), impaired glucose metabolism and energy homeostasis (*P* < 0.001), enhanced apoptotic cell death, and induced endoplasmic reticulum stress leading to autophagy (*P* < 0.001) in human CRC cell lines (HCT 116 and HT-29) (Anastasio et al., 2024). Mechanistically, these effects were mediated via the upregulation of sirtuin 3 (SIRT3), a key mitochondrial deacetylase. Transient SIRT3 silencing attenuated canagliflozin-induced metabolic alterations and programmed cell death, confirming the critical role of the SGLT-2/SIRT3 axis in the drug’s direct cytotoxic mechanisms (Anastasio et al., 2024). DPP4 was also identified as a potential common target of SGLT-2 and SIRT3 for mediating cytotoxicity (Anastasio et al., 2024).

Agent-specific differences strongly influence the mechanisms and observed effects of SGLT-2 inhibitors on CRC. Canagliflozin exerts direct cytotoxicity, cell cycle arrest, and metabolic stress via the SGLT2/SIRT3 axis in CRC cell lines (Anastasio et al., 2024). In contrast, tofogliflozin does not directly inhibit CRC cell proliferation but suppresses tumorigenesis indirectly by improving glycemia, reducing TNF-α, and alleviating chronic inflammation in diabetic obese mice (Kato et al., 2019). Clinical studies further indicate relatively consistent risk reduction across individual SGLT-2 inhibitors, without significant differential effects on CRC incidence (Suzuki et al., 2024). These direct versus indirect mechanistic differences explain the heterogeneous preclinical findings and highlight that class-wide interpretations may oversimplify agent-specific biological activities.

### 3.4 Clinical implications and future directions

The following clinical suggestions are based primarily on observational evidence and mechanistic plausibility, and should therefore be considered exploratory rather than definitive.

#### 3.4.1 Current evidence limitations and clinical implications

1. Observational Nature and Confounding: Most epidemiological evidence stems from observational studies susceptible to residual confounding factors such as lifestyle and T2DM severity (Suzuki et al., 2024; Sung et al., 2024).
2. Significant Heterogeneity: Substantial heterogeneity exists across published studies, possibly due to variations in populations, follow-up durations, and specific SGLT-2 inhibitor analogs (Hajishah et al., 2025).
3. Inconsistent Preclinical Findings: Preclinical findings regarding direct cytotoxicity are inconsistent, with agents like tofogliflozin lacking direct effects while canagliflozin exhibits them, likely reflecting differences in experimental conditions or drug-specific properties (Anastasio et al., 2024; Kato et al., 2019).
4. Lack of Causal Evidence from MR: The absence of a causal signal in Mendelian randomization analysis highlights the need for confirmatory RCTs (Ju et al., 2024).

Collectively, based on the current evidence, SGLT-2 inhibitors offer a favorable therapeutic profile for T2DM patients concerned about CRC risk:

- For Primary Prevention: In T2DM patients at high CRC risk (e.g., family history of CRC, obesity, long-standing diabetes), SGLT-2 inhibitors (e.g., dapagliflozin, empagliflozin) may represent a favorable choice compared to DPP-4 inhibitors or insulin secretagogues, given the consistent observational signal of reduced risk (Suzuki et al., 2024; Sung et al., 2024; Hajishah et al., 2025).
- For Established CRC: In T2DM patients with established CRC, SGLT-2 inhibitors may provide dual benefits of glycemic management and potential improvement in cancer outcomes, as suggested by improved survival data (Chiang et al., 2024).
- Broad-Spectrum Potential: The consistent protective signal across multiple cancer types in some studies suggests that SGLT-2 inhibitors may offer broad-spectrum metabolic benefits that extend beyond CRC-specific effects (Hajishah et al., 2025).

#### 3.4.2 Future research priorities

##### 3.4.2.1 Confirmatory RCTs

Large-scale, prospective RCTs specifically designed to evaluate CRC incidence and outcomes as primary or secondary endpoints are essential to establish causality and quantify the magnitude of benefit.

##### 3.4.2.2 Mechanistic elucidation

Studies should employ patient-derived organoids (PDOs) to compare canagliflozin (direct plus indirect effects) and tofogliflozin (indirect effects only), and to validate the SGLT-2/SIRT3 axis as a therapeutic target (Anastasio et al., 2024). Further research should clarify why agents such as tofogliflozin lack direct cytotoxicity despite demonstrating *in vivo* efficacy (Kato et al., 2019).

##### 3.4.2.3 Personalized approaches

Research should aim to identify patient subgroups (e.g., defined by obesity status, inflammatory markers, or tumor molecular subtypes) that derive maximal benefit from SGLT-2 inhibitor therapy.

##### 3.4.2.4 Combination strategies

Preclinical and clinical investigations of synergistic effects between SGLT-2 inhibitors and conventional cancer therapies, including chemotherapy and immunotherapy, are warranted.

##### 3.4.2.5 Analog-specific effects

Comparative studies of different SGLT-2 inhibitors (e.g., canagliflozin, dapagliflozin, empagliflozin, tofogliflozin) are needed to determine whether observed effects represent class-wide properties or drug-specific characteristics, which could inform agent selection.

##### 3.4.2.6 Dose-response and long-term safety

Studies should investigate dose-response relationships for SGLT-2 inhibitors (e.g., standard vs. high-dose) on CRC risk and survival, and conduct long-term (>10 years) prospective studies to confirm durability of the protective effect in at-risk populations (Hajishah et al., 2025).

## 4 GLP-1 RAs and CRC

### 4.1 Key conclusions

Glucagon-like peptide-1 receptor agonists (GLP-1 RAs) are incretin-based antidiabetic drugs that act through GLP-1R signaling to regulate glucose homeostasis and also exhibit pharmacological effects on cell proliferation and survival. Regarding CRC risk, the current high-level evidence presents a reassuring yet nuanced picture. Meta-analyses of RCTs—the gold standard for safety assessment—consistently demonstrate the absence of a class-wide increased risk of gastrointestinal cancers: one meta-analysis of 90 RCTs (124,791 participants) reported a non-significant RR for CRC (1.13, 95% CI: 0.92–1.39) with high certainty of evidence, while another RCT meta-analysis reported a marginal increased in risk (MH-OR = 1.27, 95% CI: 1.03–1.57) attributable to detection bias (Figlioli et al., 2024; Silverii et al., 2025).

Apparent risk signals identified in some observational studies are likely attributable to residual confounding and detection bias (Silverii et al., 2025). However, a distinct drug-specific and dose-dependent signal has been identified: a network meta-analysis of 68 RCTs (207,200 participants) found that high-dose injectable semaglutide (2.4 mg/week) was the only GLP-1 RA associated with a statistically significant increase in colorectal tumor incidence, requiring vigilant monitoring (Hung et al., 2025). Crucially, a fundamental translational gap exists: while GLP-1 RAs show consistent anti-tumor effects in preclinical models, these effects are largely not translatable to most human CRCs in clinical settings due to the frequent absence of the target GLP-1 receptor (GLP-1R) (Wenjing et al., 2017).

### 4.2 Epidemiological evidence: from apparent contradiction to consensus

The epidemiological data, when stratified by study design and comparator, reveals a coherent narrative that resolves apparent surface-level contradictions.

#### 4.2.1 Definitive safety signal from RCTs

RCTs, the gold standard of evidence, provide clear reassurance. A comprehensive meta-analysis of 90 RCTs involving 124,791 participants (average follow-up 3.1 years) found no significant association between GLP-1 RA use and the risk of gastrointestinal cancers, including CRC (RR = 1.13, 95% CI: 0.92–1.39). The corresponding absolute risk differences ruled out clinically meaningful impacts on risk, with high certainty of evidence (Figlioli et al., 2024). Another large RCT meta-analysis noted a marginal increase in CRC risk (MH-OR = 1.27, 95% CI: 1.03–1.57) but critically attributed this finding to detection bias—the gastrointestinal side effects (e.g., nausea, constipation) of GLP-1 RAs lead to more diagnostic colonoscopies, thereby increasing the detection of pre-existing, asymptomatic neoplasms (Silverii et al., 2025).

#### 4.2.2 Conflicting signals in observational studies and explanatory biases

However, observational studies yield highly heterogeneous results and are heavily influenced by methodology and comparator choice.

##### 4.2.2.1 Signals suggesting increased risk

Some meta-analyses of observational data have reported an association between GLP-1 RA use and increased CRC risk (RR = 2.31, 95% CI: 1.82–2.93) (Zhong et al., 2025). However, this association was rendered statistically non-significant when GLP-1 RAs were directly compared with other antidiabetic drugs (OR = 1.73, 95% CI: 0.21–14.18), highlighting the profound role of residual confounding (Zhong et al., 2025).

##### 4.2.2.2 Signals suggesting neutral or protective effects

In contrast, numerous large-scale real-world studies report neutral or potentially protective associations. A study using the UK CPRD found no association between GLP-1 RA use and CRC incidence compared to sulfonylureas (HR = 1.0, 95% CI: 0.7–1.6) (Abrahami et al., 2018). Similarly, a cohort study of US Medicare beneficiaries found no short-term effect of GLP-1 RAs on CRC risk when compared with long-acting insulin (adjusted HR = 0.82, 95% CI: 0.42–1.58) (Htoo et al., 2016).

Conversely, multiple real-world studies report protective associations: a meta-analysis of 5 observational studies found GLP-1 RAs associated with reduced CRC risk compared to thiazolidinediones (RR = 0.82, 95% CI: 0.68–0.96), insulin (RR = 0.57, 95% CI: 0.32–0.81), and SGLT-2 inhibitors (RR = 0.77, 95% CI: 0.59–0.95) (Bushi et al., 2025); a large retrospective cohort study (460,830 patients) reported a lower CRC risk with GLP-1 RAs vs. oral antidiabetics (HR = 0.870, *P* = 0.001) (Niu et al., 2025); another real-world analysis found GLP-1 RA use associated with a lower colon cancer risk vs. metformin (aOR = 0.85, *P* = 0.03) (Wang and Kim, 2022).

Notably, a real-world observational study comparing semaglutide to DPP-4 inhibitors in overweight/obese T2DM patients found semaglutide use associated with a lower CRC risk (HR = 0.80, 95% CI: 0.67–0.97) (Ipaye et al., 2025). This contrast underscores the complexity of isolating direct drug effects from the potent metabolic improvements and weight loss induced by GLP-1 RAs, as well as the influence of comparator selection, as these factors often confound causal inference.

Several factors may explain the heterogeneous findings across observational studies of GLP-1 RAs. First, comparator choice substantially influences effect estimates: when GLP-1 RAs are compared with sulfonylureas or insulin (which are associated with increased CRC risk), they appear protective; when compared with DPP-4 inhibitors or metformin, associations are neutral or only weakly protective.^68-71^ Second, confounding by weight loss is particularly relevant: GLP-1 RAs induce significant weight reduction, and obesity is an established CRC risk factor. Observational studies cannot easily disentangle direct drug effects from weight-loss-related benefits (Ipaye et al., 2025). Third, detection bias may create spurious signals: gastrointestinal side effects (nausea, constipation) lead to increased colonoscopy utilization, thereby increasing the detection of pre-existing, asymptomatic neoplasms and creating an apparent ‘increased risk’ that is not causally attributable to the drug (Silverii et al., 2025). Fourth, dose and agent specificity matters: high-dose semaglutide (2.4 mg/week for obesity) shows a distinct risk signal not observed with standard-dose GLP-1 RAs used for T2DM (Hung et al., 2025). Fifth, species-specific differences further limit translation: exenatide and liraglutide inhibit growth in murine CRC cells that express functional GLP-1R (Koehler et al., 2011; awfik and Mohamed, 2016; Li et al., 2025), but GLP-1R is frequently absent in human CRC, explaining the lack of direct anti-tumor effects in patients (Wenjing et al., 2017). Longer duration of GLP-1 RA use is associated with lower CRC risk in observational studies, suggesting a time-dependent protective trend unrelated to the short-term semaglutide signal (Wang and Kim, 2022). These diverse factors underscore that the apparent contradictions in the GLP-1 RA literature reflect methodological, population, and model-specific differences rather than biological inconsistency.

#### 4.2.3 The semaglutide exception: a dose-dependent risk signal

Emerging data highlight that CRC risk may not be uniform across the class. A network meta-analysis of 68 RCTs identified high-dose injectable semaglutide (2.4 mg/week, used for obesity) as the only GLP-1 RA associated with a statistically significant increase in colorectal tumor incidence (Hung et al., 2025). This agent-specific and dose-dependent signal mandates dedicated post-marketing surveillance.

### 4.3 Pharmacological mechanisms

Preclinical and clinical mechanistic data reveal a critical mechanistic disconnect that informs risk interpretation.

#### 4.3.1 Anti-tumor effects in preclinical models and a translational barrier

In murine models and GLP-1R-expressing rodent cell lines, agents like exenatide and liraglutide robustly inhibit CRC tumor progression through multiple pathways: exenatide suppresses 1,2-dimethylhydrazine (DMH)-induced colon carcinogenesis in diabetic mice by reducing angiogenic markers (CD34) and cell proliferation (Ki-67) (Awfik and Mohamed, 2016); liraglutide induces G1-S phase arrest and apoptosis in CRC cells, modulates the TGF-β/Smad3 signaling pathway (decreasing TGF-β and p-Smad3/Smad3, increasing E-cadherin, decreasing N-cadherin) to suppress epithelial-mesenchymal transition (EMT), and blocks the PI3K/Akt/mTOR pathway to reduce CRC cell migration and invasion (Li et al., 2025; Tong et al., 2022). These preclinical anti-tumor effects are highly dependent on the expression of functional GLP-1R in tumor cells—a prerequisite that is rarely satisfied in human clinical settings.

#### 4.3.2 Lack of GLP-1R in human CRC: the translational gap

A pivotal study using immunohistochemical analysis revealed that functional GLP-1R expression is frequently absent in both human CRC tissues and colon cancer cell lines (Wenjing et al., 2017). Consequently, exendin-4 (exenatide) exerts no modulatory effects on the growth or apoptosis of human CRC cells *in vitro* or *in vivo* (Wenjing et al., 2017). This fundamental species-specific discrepancy explains why the consistent anti-tumor effects observed in rodents have not translated into protective epidemiological signals and argues against a direct receptor-mediated carcinogenic effect of these drugs on the majority of human CRCs in clinical practice.

### 4.4 Clinical implications and future directions

The following clinical suggestions are based primarily on evidence from RCTs (for safety) and observational studies (for exploratory benefits), and should be interpreted with appropriate caution.

#### 4.4.1 Recommendations for clinical practice

##### 4.4.1.1 Overall safety

For the majority of GLP-1 RAs (e.g., liraglutide, dulaglutide, low-dose semaglutide for T2DM) in the management of T2DM and obesity, current evidence from RCTs supports a favorable safety profile with respect to gastrointestinal cancers (Figlioli et al., 2024). Their substantial cardiorenal and metabolic benefits far outweigh any unsubstantiated theoretical cancer risks.

##### 4.4.1.2 Prudence with high-dose semaglutide

Clinicians should be aware of the potential risk signal observed in RCTs for high-dose semaglutide (2.4 mg/week) indicated for obesity management (Hung et al., 2025). Prescribing decisions should take into account individual CRC risk factors (e.g., family history, personal history of polyps). This does not preclude use but suggests that such patients should adhere to age-appropriate CRC screening protocols (e.g., colonoscopy every 5–10 years) to facilitate early detection of neoplastic lesions (Hung et al., 2025).

##### 4.4.1.3 Acknowledging the protective effect of weight loss

The significant weight loss achieved with GLP-1 RAs is itself a potent factor in mitigating long-term obesity-associated CRC risk. This indirect protective effect may offset any minimal hypothetical drug-related risk.

#### 4.4.2 Future research priorities

##### 4.4.2.1 Long-term follow-up

Prioritize large-scale long-term post-marketing surveillance and extension studies of RCTs, especially for high-dose semaglutide, to assess CRC risk over the full carcinogenic latency period (10+ years) (Hung et al., 2025).

##### 4.4.2.2 Disentangling direct vs. indirect effects

Employ matched patient cohorts with similar weight loss trajectories (e.g., GLP-1 RAs vs. bariatric surgery) to isolate direct pharmacologic effects from weight loss-related benefits on CRC risk, and quantify the contribution of detection bias to observed risk signals in real-world settings (Ipaye et al., 2025).

##### 4.4.2.3 Exploring GLP-1R-independent mechanisms

Investigate whether GLP-1 RAs exert any clinically relevant effects on human CRC through off-target or indirect pathways (e.g., systemic metabolic modulation, immune regulation) (Wenjing et al., 2017).

##### 4.4.2.4 Confirmation of observational findings

Conduct large-scale prospective cohort studies with standardized comparator selection to validate the neutral or protective CRC risk signals observed in real-world databases (Bushi et al., 2025; Niu et al., 2025; Wang and Kim, 2022).

##### 4.4.2.5 Analog-specific comparative studies

Conduct head-to-head comparative studies of GLP-1 RA analogs (e.g., semaglutide vs. liraglutide) to clarify whether the observed colorectal tumor risk signal is specific to high-dose semaglutide and to identify potential risk-mitigating strategies (Hung et al., 2025).

## 5 DPP-4 inhibitors and CRC

### 5.1 Key conclusions

DPP-4 inhibitors are oral antidiabetic agents that inhibit DPP-4/CD26 enzymatic activity, with pleiotropic pharmacological effects on inflammation, immunity, and tumor progression. DPP-4 inhibitors exhibit the most controversial and inconsistent evidence profile regarding CRC risk in patients with T2DM. The literature is characterized by a fundamental methodological dichotomy: meta-analyses of RCTs frequently report neutral or potentially protective effects on CRC incidence, whereas observational studies and their meta-analyses often report signals of increased risk (Dicembrini et al., 2020; Li et al., 2022). A meta-analysis of 8 observational studies indicated that DPP-4 inhibitor exposure was associated with an 11% higher incidence of CRC (OR = 1.11, 95% CI: 1.02–1.21, *P* = 0.013), with a striking J-shaped dose-response relationship: high cumulative doses correlated with a significant 86% increased risk (adjusted OR = 1.86, 95% CI: 1.32–2.61, *P* < 0.001) (Fu et al., 2025; Chou et al., 2022). Risk is particularly elevated in males, patients under 65 years, and those with shorter exposure durations (<1 year) (Fu et al., 2025).

This contradiction critically extends to cancer prognosis, with human studies reporting both improved and worsened survival in T2DM patients with CRC who use DPP-4 inhibitors (Saito et al., 2021; Ng et al., 2021; Bishnoi et al., 2019). Mechanistically, DPP-4 (CD26) is a pleiotropic enzyme: its inhibition can promote EMT and immunosuppression (reduced CD3+/CD8+ T cells, increased M2 macrophages), or suppress inflammation and tumorigenesis (inhibit IL-6/JAK2/STAT3/NF-κB pathways), reflecting context-dependent dual biological potential (Saito et al., 2021; Yorifuji et al., 2016; Mahmoud et al., 2025). Given this unresolved controversy, the inconsistent risk profile, and the availability of alternative agents with more favorable and consistent evidence, DPP-4 inhibitors are not routinely recommended as a first-choice option for T2DM patients at high risk for CRC, thus necessitating a cautious, individualized risk-benefit assessment in clinical practice (Sung et al., 2024; Hajishah et al., 2025; Kuo et al., 2025).

### 5.2 Epidemiological evidence: a tripartite conflict

#### 5.2.1 A tripartite conflict in CRC risk association

Across study designs and populations, the association between DPP-4 inhibitor use and CRC incidence is highly heterogeneous, yielding three distinct lines of evidence.

##### 5.2.1.1 Neutral association

Multiple large-scale observational studies report neutral associations: a population-based nested case-control study of 2.08 million T2DM patients found DPP-4 inhibitor use was not associated with altered CRC risk (aOR not significant) (Shin et al., 2020); analyses of UK CPRD (HR = 1.2, 95% CI: 1.0–1.5) and US Medicare data (DPP-4i vs. sulfonylureas: HR = 0.98, 95% CI: 0.74–1.30; DPP-4i vs. thiazolidinediones: HR = 1.17, 95% CI: 0.88–1.71) also showed no significant associations (Htoo et al., 2016; Abrahami et al., 2018).

##### 5.2.1.2 Association with increased risk

A meta-analysis of 8 observational studies (6 cohort, 2 case-control) indicated that DPP-4 inhibitor exposure was associated with an 11% higher CRC incidence (OR = 1.11, 95% CI: 1.02–1.21, *P* = 0.013) (Fu et al., 2025). Subgroup analysis revealed marked population-specific differences: males (OR = 2.07, *P* < 0.001) and patients < 65 years (OR = 2.81, *P* < 0.001) had significantly higher risk, while exposure duration < 1 year also correlated with elevated risk (OR = 1.24, *P* = 0.005) (Fu et al., 2025). The J-shaped dose-response relationship shows high cumulative doses (high cDDD) associated with an 86% increased risk (adjusted OR = 1.86, 95% CI: 1.32–2.61, *P* < 0.001), whereas low cDDD may be associated with reduced risk (adjusted OR = 0.49, 95% CI: 0.32–0.75, *P* = 0.001) (Chou et al., 2022).

##### 5.2.1.3 Association with potential protection

In contrast, meta-analyses of RCTs report protective effects: a comprehensive analysis of 157 RCTs found DPP-4 inhibitors associated with a 30% reduction in CRC risk (MH-OR = 0.70, 95% CI: 0.53–0.94, *P* = 0.020) (Dicembrini et al., 2020); another meta-analysis of 115 RCTs confirmed a significant reduction in rectal neoplasms, especially rectal malignant neoplasms (OR = 0.91, 95% CI: 0.80–0.97) (Li et al., 2022).

Notably, comparative studies underscore the relative risk disadvantage of DPP-4 inhibitors: two meta-analyses found SGLT-2 inhibitors were associated with lower CRC risk compared to DPP-4 inhibitors (RR = 0.80, 95% CI: 0.70–0.94; aHR = 0.90, 95% CI: 0.87–0.93), further supporting the cautious use of DPP-4 inhibitors in high-risk patients (Sung et al., 2024; Hajishah et al., 2025).

The apparent contradiction between RCTs (suggesting protection) and observational studies (suggesting neutral or increased risk) arises from fundamental differences in study design and population. First, RCTs of DPP-4 inhibitors typically have short follow-up durations (2–5 years) and are underpowered for cancer endpoints, which require prolonged latency periods to manifest. Second, observational studies suffer from confounding by indication: DPP-4 inhibitors are preferentially prescribed to older, frailer patients with longer diabetes duration and more comorbidities—a profile independently associated with higher CRC risk. Third, the J-shaped dose-response relationship identified in observational studies implies that low doses may be neutral or protective (Chou et al., 2022), whereas high cumulative doses increase risk—a nuance that most RCTs, with fixed doses and short durations, cannot capture. Thus, the conflicting evidence reflects methodological limitations rather than biological inconsistency.

#### 5.2.2 Contradictory impact on prognosis in established CRC

The effect of DPP-4 inhibitors on outcomes for T2DM patients who develop CRC is similarly conflicted.

##### 5.2.2.1 Evidence for adverse prognosis

A key retrospective study of 260 T2DM patients after curative CRC resection found that DPP-4 inhibitor users had significantly worse 5-year disease-free survival (DFS: 73.7% vs. 87.4%; HR = 1.98, 95% CI: 1.05–3.71, *P* = 0.035) compared to non-users (Saito et al., 2021). Notably, analysis of tumor tissues suggested this adverse outcome was associated with a pro-tumorigenic immune microenvironment (fewer CD3+/CD8+ T cells, more M2 macrophages, reduced tertiary lymphoid structures) and evidence of enhanced epithelial-mesenchymal transition (EMT) (increased Zeb1+ tumor cells) (Saito et al., 2021).

##### 5.2.2.2 Evidence for beneficial prognosis

Conversely, other clinical studies demonstrate favorable prognostic effects. A single-center retrospective study found DPP-4 inhibitor users had longer disease-free survival and lower recurrence rates than patients on metformin monotherapy, identifying DPP-4 inhibitor use as an independent protective factor (HR = 0.200, *P* = 0.035) (Ng et al., 2021). A SEER-Medicare database study suggested improved OS in diabetic CRC patients using DPP-4 inhibitors (HR = 0.89, 95% CI: 0.82–0.97, *P* = 0.007), with the benefit more pronounced when combined with metformin (HR = 0.77, 95% CI: 0.67–0.89, *P* = 0.003) (Bishnoi et al., 2019).

### 5.3 Pharmacological mechanisms

DPP-4 is a multifunctional protease that cleaves incretin hormones and numerous immunomodulatory chemokines, underlying its complex, context-dependent role in cancer biology (Saito et al., 2021; Varela-Calviño et al., 2021).

#### 5.3.1 Potential anti-tumor mechanisms

Preclinical studies and network pharmacology analyses support several potential anti-tumor mechanisms.

##### 5.3.1.1 Anti-inflammatory and antioxidant effects

Specifically, in rodent models of colitis-associated CRC, DPP-4 inhibitors exert potent anti-inflammatory and antioxidant activities: linagliptin suppresses the IL-6/JAK2/STAT3/NF-κB pathway and reduces NF-κB expression (Mahmoud et al., 2025); sitagliptin downregulates IL-6 mRNA expression and reduces reactive oxygen species (ROS) levels, thereby suppressing tumorigenesis (Yorifuji et al., 2016; Femia et al., 2013). Sitagliptin also suppresses plasma CXCL5 and SDF-1 levels in high-fat diet-fed ApcMin/+ mice (Fujiwara et al., 2017).

##### 5.3.1.2 Direct cytotoxicity and pathway modulation

*In vitro*, DPP-4 inhibitors exhibit dose-dependent cytotoxicity against CRC cell lines (HT-29): sitagliptin has an IC50 of 31.2 mcg/ml, while vildagliptin has an IC50 of 125 mcg/ml (Amritha et al., 2015). They also modulate cancer-associated pathways such as PI3K-Akt and ECM-receptor interaction (Bardaweel et al., 2025). Sitagliptin can target signaling hubs centered on CD24/SOX4/CTNNB1 to inhibit cancer stem cell properties, and synergizes with 5-fluorouracil (5-FU) to suppress tumorigenicity (Shih et al., 2024).

##### 5.3.1.3 Inhibition of invasion

DPP-4 enzymatic activity contributes to the invasive capacity of CD26^+^ CRC cells, and its inhibition by sitagliptin can suppress this collagen matrix-dependent invasion. Sitagliptin also opposes TGF-β1-induced EMT and modulates cell cycle progression (Varela-Calviño et al., 2021).

#### 5.3.2 Potential pro-tumor mechanisms

##### 5.3.2.1 Impairment of immune surveillance and induction of EMT

As observed in human tumor samples, DPP-4 inhibitor use correlates with an immunosuppressive tumor microenvironment: reduced densities of CD3+/CD8+ T cells and tertiary lymphoid structures, increased M2-type (CD68^+^CD163+) macrophages, and elevated Zeb1+ tumor cells (a key EMT transcription factor), suggesting that DPP-4 inhibition may accelerate this pro-metastatic process (Saito et al., 2021).

##### 5.3.2.2 Modulation of chemokine signaling

In animal models, DPP-4 inhibition can alter levels of chemokines like CXCL5 and SDF-1, which may affect immune cell recruitment and tumor-stroma interactions (Fujiwara et al., 2017).

### 5.4 Clinical implications and future directions

The following clinical suggestions are based on highly inconsistent evidence (RCTs suggesting protection versus observational studies suggesting neutral or increased risk). Readers should therefore interpret these recommendations as precautionary and hypothesis-generating, pending confirmation from better-designed studies.

#### 5.4.1 Implications for clinical decision-making

##### 5.4.1.1 Risk-benefit assessment and drug selection

Given the unresolved and contradictory evidence, DPP-4 inhibitors should not be the preferred therapeutic option for T2DM patients at high CRC risk (e.g., first-degree relative diagnosed with CRC at age <50 years, personal history of advanced adenomas, male sex, age <65 years) (Fu et al., 2025). Alternative glucose-lowering agents with more consistently favorable CRC risk profiles (e.g., SGLT-2 inhibitors, GLP-1 RAs) are preferable (Sung et al., 2024; Hajishah et al., 2025; Kuo et al., 2025).

##### 5.4.1.2 Considerations for dose and combination therapy

The identified J-shaped dose-response relationship suggests that if DPP-4 inhibitor use is necessary, lower to moderate cumulative doses might be preferable over high-dose, long-term therapy (Chou et al., 2022). Combination with metformin may not only improve glycemic control but may also mitigate adverse prognostic effects, as suggested by some survival data (HR = 0.77, 95% CI: 0.67–0.89, *P* = 0.003) (Bishnoi et al., 2019).

##### 5.4.1.3 Monitoring and screening

For patients prescribed DPP-4 inhibitors, strict adherence to standard age-appropriate CRC screening guidelines is paramount. Current evidence does not support more intensive screening solely based on DPP-4 inhibitor use.

#### 5.4.2 Limitations of current evidence

##### 5.4.2.1 Study design discrepancy

RCTs, while high-quality for efficacy, are typically short-term (2–5 years) and underpowered to detect effects on long-latency outcomes like cancer incidence. Observational studies, capable of longer follow-up, are severely susceptible to residual confounding (e.g., by indication, lifestyle, unmeasured comorbidities).

##### 5.4.2.2 Confounding by indication

DPP-4 inhibitors are often prescribed to older, frailer patients with longer diabetes duration and more advanced disease—a profile inherently associated with higher baseline cancer risk and mortality, which can create a spurious signal of harm.

##### 5.4.2.3 Insufficient long-term data

The long latency period of CRC means most studies, especially RCTs, have inadequate follow-up duration to conclusively assess risk.

#### 5.4.3 Future research priorities

##### 5.4.3.1 Long-term, prospective, comparative effectiveness studies

There is an urgent need for large, well-designed prospective cohorts or pragmatic trials with extended follow-up (>10 years), conducting head-to-head comparisons of DPP-4 inhibitors with SGLT-2 inhibitors or GLP-1 RAs. Primary endpoints should include CRC incidence and disease-free survival, with stratification by sex, age, and cumulative dose (Chou et al., 2022).

##### 5.4.3.2 Personalized medicine and biomarker discovery

Research must identify predictive biomarkers (e.g., tumor CD26/DPP-4 expression, specific immune cell profiles, germline genetics) to determine which patient subgroups might derive benefit or be at risk from DPP-4 inhibitor therapy, thereby enabling personalized therapeutic decision-making.

##### 5.4.3.3 Mechanism-to-outcome translational research

Studies that integrate deep molecular profiling of tumor and immune microenvironment with detailed clinical outcomes in DPP-4 inhibitor users are crucial to validate the proposed immunemodulatory and EMT-mediated mechanisms observed in preclinical and retrospective human studies (Saito et al., 2021).

##### 5.4.3.4 Analog-specific effect investigations

Analog-specific effects (e.g., sitagliptin vs. linagliptin) on CRC risk and mechanisms should be investigated, as preclinical data suggest variable potency (Amritha et al., 2015).

## 6 Insulin and CRC

### 6.1 Key conclusions

Exogenous insulin is a core glucose-lowering therapy that exerts pharmacological effects by activating insulin/IGF-1 signaling, which is closely linked to cancer cell proliferation and survival. A substantial body of evidence from meta-analyses, observational studies, and Mendelian randomization analyses consistently support an association between insulin therapy and an increased risk of CRC and its precursor lesions (adenomas) in T2DM patients. Multiple meta-analyses consistently confirm this causal link: insulin use is associated with a 37%–69% increased CRC risk (RR range: 1.37–1.69), with a landmark meta-analysis reporting a 50% elevation (RR = 1.50, 95% CI: 1.08–2.08). (Sun et al., 2014; Bu et al., 2014; Janghorbani et al., 2012). This risk exhibits clear dose- and duration-dependence, is more pronounced in specific populations (e.g., Asian and American cohorts), and is linked to worse survival outcomes in patients with established CRC.

Importantly, the pro-carcinogenic effect of insulin appears to be modifiable, as concurrent use of metformin may attenuate the excess risk (Currie et al., 2009). The evidence regarding long-acting insulin analogs (e.g., insulin glargine) is conflicting: clinical studies report neutral or even reduced CRC risk (RR = 0.78, 95% CI: 0.64–0.94), while *in vitro* studies show high-dose glargine (150–300 nM) promotes CRC cell proliferation, highlighting the need for formulation-specific evaluation (Colmers et al., 2012; Qin et al., 2015).

### 6.2 Epidemiological and clinical outcome evidence

#### 6.2.1 Consistent findings from meta-analyses

Multiple meta-analyses of observational studies provide consistent evidence that insulin therapy is associated with an increased risk of CRC in patients with T2DM. Foundational meta-analyses incorporating 12 observational studies (7 case-control, 5 cohort) reported a pooled RR of 1.69 (95% CI: 1.25–2.27) for CRC among insulin users, corroborated by another meta-analysis of 4 studies (Bu et al., 2014; Yin et al., 2014; Wang et al., 2013). This harmful association has been validated and updated by subsequent meta-analyses, with reported risk increases ranging from 37% to 61% (Sun et al., 2014; Janghorbani et al., 2012; Wang et al., 2013).

#### 6.2.2 Genetic evidence and sources of heterogeneity

Higher-level genetic evidence supports a causal link. A two-sample Mendelian randomization study (8 SNPs as instrumental variables) confirmed that genetically predicted insulin use was associated with an 11% increased risk of CRC (OR = 1.104, 95% CI: 1.018–1.196, *P* = 0.016), minimizing concerns about confounding by indication (Chen et al., 2025).

The strength of the observed association exhibits significant heterogeneity. The risk is markedly stronger in case-control studies (RR = 2.15, 95% CI: 1.41–3.26) than in cohort studies (RR = 1.25, 95% CI: 0.95–1.65), and is most pronounced in Asian (RR = 2.55, 95% CI: 2.14–3.04) and US populations (RR = 1.73, 95% CI: 1.15–2.60), while European cohorts show non-significant associations (RR = 1.20, 95% CI: 0.92–1.57) (Bu et al., 2014).

Crucially, the risk demonstrates a clear dose- and duration-dependent relationship. Long-term insulin use (≥1 year) is associated with a 2.1-fold increased risk of CRC compared to non-use (HR = 2.1, 95% CI: 1.2–3.4, *P* = 0.005), with each additional year of therapy increasing CRC risk by 21% (OR = 1.21, 95% CI: 1.03–1.42, *P* = 0.02) (Yang et al., 2004). This extends to CRC precursors: chronic insulin use (≥1 year) is linked to a 3-fold higher risk of colorectal adenomas (OR = 3.0, 95% CI: 1.1–8.9, *P* = 0.04) and a substantially increased risk of advanced adenomas (adjusted HR up to 5.48 for right-sided lesions) (Chung et al., 2008; Lam et al., 2024). A population-based cohort study further validated this: insulin use was associated with an 86% increased CRC risk (aHR = 1.86, 95% CI: 1.58–2.19) compared to non-insulin users among T2DM patients (Chen et al., 2020).

Dose-response and insulin-type differences are key sources of heterogeneous CRC risk outcomes. Longer duration and higher cumulative insulin exposure are associated with progressively greater CRC and advanced adenoma risk, consistent with a sustained mitogenic effect of chronic hyperinsulinemia (Yang et al., 2004; Chung et al., 2008; Lam et al., 2024; Chen et al., 2020). For insulin type, human insulin and long-acting analogs (e.g., glargine) differ in biological activity: high-dose glargine directly promotes CRC cell proliferation *in vitro* (Qin et al., 2015), yet clinical studies show no increased CRC risk with glargine compared with human insulin (Colmers et al., 2012; Pradhan et al., 2020; Habel et al., 2013). This discrepancy likely reflects differences between suprapharmacological *in vitro* doses and physiological therapeutic exposure in humans. These dose- and formulation-specific effects explain the variable risk estimates across studies.

#### 6.2.3 Impact on clinical outcomes and risk modulation with metformin

Beyond carcinogenesis, insulin use adversely affects prognosis in patients with established CRC. A population-based cohort study found that ever use of insulin was associated with significantly worse OS in CRC patients (HR = 1.89, 95% CI: 1.57–2.29) (Baglia et al., 2019).

Importantly, the pro-carcinogenic risk associated with insulin appears modifiable through combination therapy. While insulin increases CRC risk, metformin use is consistently associated with risk reduction (aHR = 0.65, 95% CI: 0.54–0.77) (Chen et al., 2020). Crucially, adding metformin to insulin therapy can mitigate the excess risk. One study showed that while insulin-based regimens carried a higher CRC risk versus metformin monotherapy (HR = 1.69, 95% CI: 1.23–2.33), concomitant metformin use reduced this risk by nearly half (HR = 0.54, 95% CI: 0.43–0.66) (Currie et al., 2009). A meta-analysis further supports an 11% reduction in CRC risk with metformin use (OR = 0.89, 95% CI: 0.81–0.99). (Singh et al., 2013).

### 6.3 Pharmacological mechanisms

#### 6.3.1 Core mechanism: hyperinsulinemia and IGF-1 pathway activation

The primary mechanistic driver is the induction of systemic hyperinsulinemia. Exogenous insulin elevates circulating insulin levels, which activate insulin receptors (IR) and insulin-like growth factor-1 receptors (IGF-1R) on colonic epithelial/CRC cells, triggering downstream pro-survival and proliferative signaling cascades—primarily the PI3K/AKT/mTOR and MAPK pathways. This promotes tumor growth, inhibits apoptosis, and stimulates angiogenesis (Qin et al., 2015). Notably, high-dose insulin glargine (150–300 nM) specifically upregulates oncogenic miR-95 and downregulates tumor suppressor SNX1, promoting CRC cell proliferation and inhibiting apoptosis *in vitro* (HCT-116, SW480 cells) (Qin et al., 2015). These findings highlight the biological plausibility of the clinical association between insulin therapy and increased CRC risk.

#### 6.3.2 The controversy surrounding long-acting analogs

The risk profile of long-acting insulin analogs, particularly insulin glargine, remains contentious. Several large clinical database studies have reported neutral or potentially protective effects on CRC risk compared to human insulin. A population-based cohort study using the UK CPRD database found that use of long-acting insulin analogs was not associated with an overall increased risk of CRC compared with intermediate-acting human insulin (HR = 0.96, 95% CI: 0.70–1.34) (Pradhan et al., 2020). Similarly, a large cohort study from Kaiser Permanente found no increased risk of CRC associated with ever use or long-term use (≥2 years) of insulin glargine compared to NPH insulin among initiators of insulin therapy (Habel et al., 2013). Another meta-analysis showed that new use of insulin glargine was associated with a decreased risk of CRC (RR = 0.78, 95% CI: 0.64–0.94, I^2^ = 15%) (Colmers et al., 2012). A comprehensive review also indicated that glargine versus non-glargine insulin was associated with a decreased risk for colon cancer (Karlstad et al., 2013).

Contrasting with *in vitro* findings, this clinical evidence creates a paradox. The discrepancy may stem from differences in dosage (pharmacological *in vitro* vs. physiological *in vivo*), exposure time, or unaccounted patient factors in clinical settings (e.g., concurrent metformin use), warranting cautious interpretation and further study.

### 6.4 Clinical implications and future perspectives

The following clinical suggestions are based on consistent observational evidence and Mendelian randomization studies supporting a causal link. While the evidence for harm is stronger than for other drug classes, recommendations remain exploratory pending confirmatory RCTs.

#### 6.4.1 Recommendations for clinical practice

Risk Awareness and Enhanced Screening: For patients on long-term insulin (≥1 year) or with additional risk factors (family history of CRC, obesity, advanced adenoma history), adherence to age-appropriate CRC screening—with consideration of earlier initiation (≥45 years) and shorter intervals (every 5 years for colonoscopy)—is paramount (Chung et al., 2008; Lam et al., 2024).

Optimization of Treatment Strategy:

1. Prioritize and maximize the use of non-insulin agents with better safety profiles (e.g., metformin, SGLT-2 inhibitors, GLP-1 RAs) to delay or reduce insulin requirements.
2. When insulin is necessary, co-prescribing metformin may be considered not only for glycemic synergy but also for its potential to mitigate cancer risk (Currie et al., 2009).
3. While evidence is not conclusive, the current clinical data do not indicate that long-acting analogs confer a higher CRC risk than human insulin. The choice should be based on glycemic control, hypoglycemia risk, and patient preference, while acknowledging the unresolved mechanistic concerns for high-dose glargine (Qin et al., 2015).

#### 6.4.2 Future research priorities

1. Large-scale, prospective cohorts with detailed data on insulin type (glargine vs. NPH), dose (therapeutic vs. high-dose), and duration are needed to clarify analog-specific risks (Pradhan et al., 2020). Studies should also resolve the in vitro-in vivo discrepancy for glargine, focusing on physiological dose effects (Qin et al., 2015).
2. Well-designed *in vivo* studies using physiologically relevant doses are required to resolve the controversy surrounding long-acting insulin analogs and to fully elucidate the downstream effectors of insulin/IGF-1 signaling in CRC.
3. RCTs exploring whether specific insulin regimens (e.g., basal-only vs. basal-bolus) or combination therapies (e.g., insulin + SGLT-2 inhibitors) differentially impact CRC risk or surrogate biomarkers (e.g., serum IGF-1, miR-95) are warranted.
4. Validate predictive biomarkers (e.g., serum IGF-1 levels, tumor miR-95/SNX1 expression) to identify T2DM patients on insulin at highest CRC risk (Qin et al., 2015).

## 7 Thiazolidinediones (TZDs) and CRC

### 7.1 Key conclusions

TZDs are PPARγ agonists with potent insulin-sensitizing pharmacological effects and additional regulatory activities on cancer cell growth and immunity. Epidemiological evidence, particularly from large Asian cohorts, demonstrates a modest yet statistically significant protective association between TZD use and reduced CRC risk in patients with T2DM (pooled RR = 0.91, 95% CI: 0.84–0.99) (Liu et al., 2018). This effect is primarily driven by rosiglitazone, while pioglitazone shows no significant protective effect (RR = 0.95, 95% CI: 0.89–1.01) (Liu et al., 2018). However, this signal is tempered by significant geographical heterogeneity (stronger in Asians vs. neutral in Western populations), agent-specific differences (pioglitazone’s bladder cancer risk), and a paradoxical duality in preclinical findings (anti- vs. pro-tumorigenic effects). While TZDs demonstrate compelling anti-tumor mechanisms *in vitro* and synergy with conventional therapies, certain *in vivo* models—such as troglitazone-induced tumor formation in wild-type mice—raise a notable concern regarding the potential for context-dependent effects that warrant further investigation (Yang et al., 2005). Therefore, TZDs should not be prescribed solely for CRC chemoprevention. However, their repurposing potential as adjunctive therapy in established CRC—supported by preclinical synergy with chemotherapy (5-fluorouracil [5-FU]) and immunotherapy (PD-1 inhibitors)—warrants serious investigation (Jia et al., 2023; Lau et al., 2020; Zhang et al., 2007).

### 7.2 Epidemiological evidence: protective association with critical heterogeneity

#### 7.2.1 Evidence supporting risk reduction

A growing body of observational data suggests that TZDs are associated with a favorable trend towards reduced CRC risk among antidiabetic drugs. A landmark 6-year population-based cohort study reported that TZD users had a 51%–61% lower overall cancer risk compared to non-users or users of other antidiabetic agents (adjusted HR = 0.39, 95% CI: 0.33–0.45, *P* < 0.001), with a clear dose-dependent trend (P for trend < 0.001) and a significant protective effect specifically for CRC (Lin et al., 2014). This finding is reinforced by multiple case-control studies, consistently reporting adjusted odds ratios (aORs) for CRC between 0.86 and 0.94 among TZD users (Chen et al., 2013; Liao et al., 2019).

A meta-analysis of 10 observational studies further confirmed this protective effect: TZD use was associated with a 9% overall reduction in CRC risk (RR = 0.91, 95% CI: 0.84–0.99, *P* = 0.03), with striking geographic heterogeneity—Asian populations showed a 60% reduction (RR = 0.40, 95% CI: 0.29–0.53), while US populations only showed a non-significant trend (RR = 0.94, 95% CI: 0.88–1.01, *P* = 0.08) (Liu et al., 2018).

#### 7.2.2 Notable heterogeneity: geographic and agent-specific disparities

##### 7.2.2.1 Geographic heterogeneity

In contrast to Asian cohorts, Western cohorts show null associations. A US nested case-control study found no significant association between TZD use and colon cancer risk (OR = 1.03, 95% CI: 0.80–1.32), and a cohort study reported no statistically significant reduction in CRC risk with TZD use (adjusted RR not significant) (Koro et al., 2007; Govindarajan et al., 2007). This heterogeneity may reflect genetic differences, dietary patterns, or variations in T2DM phenotypes.

##### 7.2.2.2 Agent-specific differences

Emerging data suggest that the observed CRC risk reduction may be primarily driven by rosiglitazone, whereas pioglitazone often shows a neutral effect on CRC (RR = 0.95, 95% CI: 0.89–1.01, *P* = 0.11) (Liu et al., 2018). Notably, pioglitazone is associated with a modestly increased risk of bladder cancer (RR = 1.20, 95% CI: 1.07–1.34), particularly with long-term, high-dose use, complicating its overall risk-benefit profile (Bosetti et al., 2013).

### 7.3 Pharmacological mechanisms

#### 7.3.1 Established anti-tumor mechanisms

A robust preclinical rationale supports the potential anti-CRC effects of TZDs. Multiple TZDs (e.g., troglitazone, ciglitazone, pioglitazone, rosiglitazone) inhibit the proliferation of human CRC cell lines by inducing G1-phase cell cycle arrest (via degradation of cyclin D1 and CDK4, and upregulation of cyclin-dependent kinase inhibitors p21 and p27) and promoting apoptosis (Yoon et al., 2020; Ming et al., 2006). This effect is mediated through modulation of Bcl-2 family proteins (downregulation of Bcl-2, upregulation of Bax) (Zhang et al., 2007).

Troglitazone also inhibited azoxymethane (AOM) or dextran sodium sulfate (DSS)-induced aberrant crypt foci (ACF) formation in rats, which was associated with increased apoptosis and reduced colonic mucosal polyamine levels and ornithine decarboxylase (ODC) activity (Kohno et al., 2001). Furthermore, TZDs suppress epithelial-mesenchymal transition (EMT)—a key driver of metastasis—by modulating the E-cadherin/β-catenin system and downregulating mesenchymal markers like vimentin (Alamdar et al., 2025; Yoshizumi et al., 2004). Pioglitazone and cetuximab can reduce the expression of CSC markers (CD133, CD44) and mesenchymal markers (vimentin) in EMT-induced CRC cells, promoting mesenchymal-to-epithelial transition (MET) (Alamdar et al., 2025).

#### 7.3.2 Synergy with conventional therapy

A significant translational finding is the synergistic potential of TZDs with standard chemotherapy. They enhance the apoptotic effect of 5-FU in CRC cells: rosiglitazone dose-dependently increases 5-FU-induced apoptosis by 5%–20% under both normal and high-glucose conditions, overcoming high-glucose-induced chemoresistance mediated by reduced glutathione (Lau et al., 2020; Zhang et al., 2007). This synergy is PPARγ-dependent, as it is blocked by the PPARγ antagonist GW9662 (Zhang et al., 2007). This synergy extends to other modalities, such as photodynamic therapy (PDT), where troglitazone enhances the apoptotic response of DLD-1 colon cancer cells to PDT by activating caspase-3 (Park et al., 2016).

#### 7.3.3 Emerging immunomodulatory role

Recent studies reveal a novel mechanism relevant to modern oncology. PPARγ agonists like pioglitazone exert immunomodulatory effects by inducing autophagic degradation of PD-L1 (without affecting PD-L1 gene expression) and promoting ubiquitination/degradation of c-Myc (Jia et al., 2023; Xu et al., 2023). c-Myc increases PD-L1 and CD47 expression to promote tumor immune escape, which is inhibited by pioglitazone (Xu et al., 2023). The combination of pioglitazone and PD-1 antibody enhances colorectal tumor immunotherapy, associated with reduced PD-L1 levels and increased CD8^+^ T cell infiltration (Jia et al., 2023).

#### 7.3.4 A note of caution: potential pro-tumorigenic effects

Contrary to the predominant anti-tumor narrative, some preclinical evidence warrants careful consideration. PPARγ activation can promote proliferation of some CRC cell lines (e.g., SW403, HT29), with high PPARγ expression associated with pulmonary metastasis and shorter OS in CRC patients without distant metastases (Schöckel et al., 2024). More importantly, a concerning *in vivo* study found that the TZD troglitazone not only enhanced colorectal carcinogenesis in Apc1638N/+ Mlh1^+/−^double mutant mice but also induced colonic tumors in wild-type C57BL/6J mice without preexisting mutational events or carcinogen administration (Yang et al., 2005). This suggests that PPARγ activation alone may, under specific conditions, be sufficient to initiate tumorigenesis.

However, human data do not support an increased risk. A retrospective study in diabetic patients found thiazolidinedione therapy was not associated with an increased risk of colonic neoplasia. While an inverse association was observed in one subgroup of patients undergoing colonoscopy, this was not consistent across all analyses, underscoring the need for cautious interpretation (Lewis et al., 2008). This critical finding highlights the complex, context-dependent, and incompletely understood nature of PPARγ signaling in colorectal carcinogenesis.

### 7.4 Clinical implications and future perspectives

The following clinical suggestions are based primarily on observational studies (particularly from Asian cohorts) and preclinical mechanistic data, and should therefore be considered exploratory rather than definitive.

#### 7.4.1 Positioning in T2DM management

For T2DM patients at high CRC risk (e.g., strong family history of CRC, personal history of advanced adenomas, Asian ethnicity), selecting rosiglitazone (where available and clinically appropriate) may offer a favorable metabolic profile with a potential ancillary oncological benefit compared to insulin or most insulin secretagogues (Liu et al., 2018; Lin et al., 2014). However, this must be balanced against individual cardiovascular risk profiles (for rosiglitazone) and bladder cancer risk (for pioglitazone) (Bosetti et al., 2013). TZDs should not be prescribed solely for CRC chemoprevention outside of clinical trials.

#### 7.4.2 Drug repurposing: potential in oncology combination therapy

The most promising future for TZDs may lie in oncology drug repurposing. Their ability to sensitize tumors to chemotherapy (5-FU), suppress cancer stem cell properties, and modulate the immune microenvironment (reduce PD-L1, increase CD8^+^ T cells) presents a compelling rationale for their integration into CRC treatment regimens (Jia et al., 2023; Lau et al., 2020; Zhang et al., 2007; Alamdar et al., 2025).

#### 7.4.3 Future research priorities

1. Elucidate the molecular determinants underlying the contrasting effects of PPARγ activation (anti- vs. pro-tumorigenic) in different genetic (e.g., p53 status) and microenvironmental (e.g., glucose levels, inflammatory status) contexts, particularly why PPARγ activation promotes proliferation in some CRC cell lines but inhibits it in others (Ming et al., 2006; Schöckel et al., 2024).
2. Conduct large-scale, prospective studies or RCTs in diverse populations to clarify the causal relationship, especially for rosiglitazone.
3. Develop predictive biomarkers (e.g., tumor PPARγ expression level, c-Myc status, PD-L1 expression) to identify patient subgroups most likely to benefit from TZD-based combination therapy (Jia et al., 2023; Xu et al., 2023).
4. Explore TZD-based combination therapies in clinical trials, including combinations with 5-FU-based chemotherapy and PD-1/PD-L1 inhibitors, to validate preclinical synergies (Jia et al., 2023; Lau et al., 2020; Zhang et al., 2007).

## 8 Insulin secretagogues and CRC

### 8.1 Key conclusions

Insulin secretagogues are oral antidiabetic drugs that promote insulin secretion via pancreatic β-cell stimulation, with divergent pharmacological impacts on CRC risk. A primary concern regarding their long-term use is a potential increase in CRC risk, largely attributed to induction of hyperinsulinemia—an established driver of CRC via insulin/IGF-1 pathway activation. However, the most striking feature of this drug class is profound analog-specific heterogeneity in CRC risk: most sulfonylureas (e.g., glimepiride) and meglitinides are associated with increased CRC risk (aOR range: 1.08–2.35), while gliclazide consistently demonstrates a protective effect (aOR = 0.85, 95% CI: 0.72–1.00) (Shin et al., 2020; Rezazadeh et al., 2024; Chang et al., 2012). This analog-specific heterogeneity invalidates a uniform class effect and necessitates a medication selection strategy based on individual CRC risk profiles in T2DM patients.

### 8.2 Epidemiological evidence: overall risk and critical heterogeneity

#### 8.2.1 Overall risk signal: sulfonylureas and meglitinides

Epidemiological studies generally associate insulin secretagogue use with an elevated CRC risk in T2DM patients, and this risk exhibits clear dose-response and age-specific patterns.

Sulfonylureas: A large population-based nested case-control study involving 2.08 million T2DM patients found that sulfonylurea use was associated with a 14% increased CRC risk (aOR = 1.14, 95% CI: 1.05–1.25), with a significant dose-response trend (Shin et al., 2020). This finding is corroborated by a case-control study of 684 CRC cases, which reported a more pronounced 2.35-fold increased CRC risk with sulfonylurea use (adjusted aOR = 2.35, 95% CI: 1.12–4.91) (Rezazadeh et al., 2024). Notably, the increased risk was confined to patients aged ≥65 years in subgroup analysis, highlighting age-related susceptibility (Shin et al., 2020). A retrospective cohort study of 88,713 US veterans further confirmed the relative disadvantage of sulfonylureas: compared to metformin users, sulfonylurea users had a significantly higher CRC risk (adjusted HR for metformin vs. sulfonylurea = 0.75, 95% CI: 0.62–0.92) (Abdallah et al., 2024).

Generational differences among sulfonylureas are also evident: a cohort study revealed that first- and second-generation sulfonylureas were associated with an 8% increased CRC risk (OR = 1.08, 95% CI: 1.01–1.15), while the third-generation drug glimepiride showed no significant association (OR = 1.00, 95% CI: 0.93–1.08) (Chang et al., 2012). A meta-analysis further confirmed that sulfonylureas were not associated with a significant change in CRC risk, in contrast to metformin (RR = 0.64, 95% CI: 0.54–0.76) (Soranna et al., 2012).

Meglitinides: A cohort study also linked glinide use to a 16% higher overall cancer risk (OR = 1.16, 95% CI: 1.06–1.28), with specific associations noted for CRC, among other cancers (Chang et al., 2012).

#### 8.2.2 Critical heterogeneity: the protective exception of gliclazide

In sharp contrast to the class trend, the second-generation sulfonylurea gliclazide demonstrates a consistent protective profile. Notably, the same population-based study identified gliclazide as an exception: its use was associated with a 15% reduced CRC risk (aOR = 0.85, 95% CI: 0.72–1.00, *P* < 0.05), distinguishing it from other sulfonylureas and supporting its consideration as the preferred agent within this class for patients at elevated CRC risk (Shin et al., 2020).

### 8.3 Pharmacological mechanisms

The divergent CRC risk profiles among insulin secretagogues are underpinned by distinct molecular mechanisms.

#### 8.3.1 Risk-associated analogs: hyperinsulinemia-driven pathway

For most sulfonylureas (e.g., glimepiride) and meglitinides, the primary postulated mechanism is sustained hyperinsulinemia. By promoting endogenous insulin secretion, these agents chronically elevate circulating insulin levels, which activates insulin and IGF-1 receptors on colonic epithelial cells. This activation promotes cell proliferation, inhibits apoptosis, and stimulates angiogenesis—key hallmarks of cancer initiation and progression (Chang et al., 2012; Bowker et al., 2006). The observed dose-response relationship in clinical studies supports this mechanism, as higher doses induce more severe hyperinsulinemia.

#### 8.3.2 The protective exception: gliclazide’s AMPK/NF-κb anti-inflammatory pathway

The protective effect of gliclazide is mediated through a mechanism largely independent of hyperinsulinemia. Preclinical studies using an AOM-DSS colitis-associated CRC mouse model confirmed that gliclazide (6 mg/kg, gavaged 5 days per week for 12 weeks) significantly reduced tumor number and burden via the AMPK-NF-κB signaling axis (Li S. et al., 2024):

1. AMPK Activation: It increases phosphorylation of AMP-activated protein kinase (p-AMPK), a key cellular energy sensor;
2. NF-κB Inhibition: Activated p-AMPK directly suppresses the NF-κB pathway (a master regulator of inflammation);
3. Anti-inflammatory and Anti-proliferative Effects: This modulation reduces pro-inflammatory cytokine TNF-α levels, increases anti-inflammatory cytokine IL-10 levels, and decreases colonic epithelial cell proliferation (evidenced by reduced Ki-67 expression), ultimately lowering tumor number and burden (Li S. et al., 2024).

This anti-inflammatory and anti-proliferative mechanism explains gliclazide’s chemopreventive potential, particularly in inflammation-driven CRC models like colitis-associated cancer.

#### 8.3.3 A unique case: glibenclamide and the NLRP3 inflammasome

Glibenclamide (glyburide), a first-generation sulfonylurea, presents a unique mechanism: it acts as an off-target NLRP3 inflammasome inhibitor (Maeda et al., 2024). In AOM-DSS-induced colitis-associated CRC mice, glibenclamide suppressed tumorigenesis by attenuating NLRP3-mediated chronic inflammation and reducing colonic inflammatory cytokine expression (Maeda et al., 2024). However, *in vitro* studies (HCT-116 and HCT-15 cells) showed it had limited direct effects on CRC cell growth, apoptosis, or invasion, suggesting its primary role is preventive in inflammation-driven carcinogenesis rather than therapeutic against established tumors (Hamza et al., 2024). This suggests glibenclamide’s activity may be primarily preventive in inflammation-initiated carcinogenesis rather than therapeutic against established tumors, distinguishing its mechanism from both hyperinsulinemia-driven agents and gliclazide.

### 8.4 Clinical implications and future perspectives

The following clinical suggestions are based on observational studies with substantial analog-specific heterogeneity. Recommendations should be considered exploratory and hypothesis-generating, particularly regarding the protective exception of gliclazide.

#### 8.4.1 Personalized therapeutic decision-making

1. Based on current evidence, for T2DM patients at high risk for CRC (e.g., personal/family history, inflammatory bowel disease), if an insulin secretagogue is indicated, gliclazide may be preferred based on its favorable risk profile.
2. Other sulfonylureas (e.g., glimepiride) and meglitinides should be used with caution in such patients, given their association with increased risk. Their use should be justified by strong individual glycemic needs and in the absence of safer alternatives (e.g., metformin, SGLT-2 inhibitors, GLP-1 RAs).
3. The age-dependent risk (≥65 years) for most sulfonylureas calls for heightened vigilance and possibly intensified CRC screening in elderly patients on these medications.

#### 8.4.2 Future research priorities

##### 8.4.2.1 Clinical studies

Large-scale, prospective studies are needed to confirm the long-term effects of specific analogs, especially gliclazide, on CRC incidence and, crucially, on prognosis in patients with established CRC.

##### 8.4.2.2 Mechanistic studies

Further research should elucidate the precise molecular divergences between protective (gliclazide) and risk-associated (glimepiride) sulfonylureas. The interplay between metabolic (insulin/IGF-1) and inflammatory (AMPK/NF-κB) pathways represents a key area for investigation.

##### 8.4.2.3 Exploration of combination therapies

The anti-inflammatory properties of gliclazide warrant investigation in combination with standard therapies for colitis-associated CRC or in patients with a pro-inflammatory metabolic phenotype.

## 9 Alpha-glucosidase inhibitors (AGIs) and CRC

### 9.1 Key conclusions

AGIs are gastrointestinal glucose-lowering agents that exert indirect pharmacological effects on metabolic disorders and colorectal tumorigenesis. Current evidence, primarily from East Asian observational studies, consistently demonstrates a favorable and dose-dependent protective association between alpha-glucosidase inhibitor (AGI) use and reduced risk of colorectal neoplasia (from precancerous adenomas to invasive cancer) in patients with T2DM. Acarbose, a representative AGI, is associated with a 27% overall reduction in incident CRC risk, with a graded protective effect by cumulative dose (Tseng et al., 2015). Representative AGIs including acarbose and voglibose have been the focus of key epidemiological and preclinical investigations, supporting their chemopreventive potential (Tseng et al., 2015; Kato et al., 2020). This association is strengthened by a clear dose-response relationship, reinforcing the argument for a potential causal relationship.

### 9.2 Epidemiological evidence: a dose-dependent protective signal

#### 9.2.1 Reduced risk of incident CRC

A cohort study involving nearly 200,000 propensity score-matched pairs of T2DM patients, demonstrated that acarbose use was associated with a significant 27% reduction in the risk of incident CRC compared to non-users (Tseng et al., 2015). Crucially, this protective effect exhibited a strong, graded dose-response relationship. The adjusted hazard ratios (HRs) were 0.73 (95% CI: 0.63–0.83), 0.69 (95% CI: 0.59–0.82), and 0.46 (95% CI: 0.37–0.58) for low (>0 to <90 cumulative defined daily doses [cDDDs]), medium (90–364 cDDDs), and high (≥365 cDDDs) exposure categories, respectively (*P* for trend <0.001) (Tseng et al., 2015). A cDDD was defined as the average daily dose of acarbose used for T2DM treatment in clinical practice.

#### 9.2.2 Protection against precancerous adenomas

The beneficial association extends to earlier stages of the adenoma-carcinoma sequence. A retrospective cross-sectional study of elderly T2DM patients (≥60 years) found that AGI use was associated with a 60.1% reduction in the risk of colorectal adenoma (adjusted OR = 0.399, 95% CI: 0.22–0.723), a finding robust to propensity score matching (PSM) analysis (post-PSM OR = 0.362, 95% CI: 0.176–0.744, *P* = 0.004) (Xia et al., 2025). Notably, subgroup analysis of this study suggested hypertension as a potential effect modifier, with a more pronounced protective effect of AGIs against colorectal adenoma observed in hypertensive patients (*P* = 0.049) (Xia et al., 2025). Additionally, AGI use was significantly associated with reduced serum iron levels both before (*P* = 0.01) and after PSM (*P* = 0.028), which may contribute to the observed risk reduction by mitigating colonic oxidative stress (Xia et al., 2025).

Similarly, a retrospective endoscopic study identified AGI use as an independent factor associated with a decreased risk of colorectal neoplasia (OR = 0.35, 95% CI: 0.13–0.87, *P* = 0.023) (Horibe et al., 2017).

### 9.3 Pharmacological mechanisms

Preclinical and clinical data converge to suggest that the chemopreventive effects of AGIs are primarily host-mediated and indirect, stemming from the correction of diabetes-associated metabolic perturbations (e.g., hyperglycemia, insulin resistance, oxidative stress) that promote carcinogenesis (Kato et al., 2020; Xia et al., 2025).

#### 9.3.1 Support from preclinical models

In an *in vivo* model relevant to diabetes-associated cancer, the AGI voglibose significantly suppressed the development of azoxymethane-induced colonic preneoplastic lesions in obese and diabetic (db/db) mice (Kato et al., 2020). Notably, voglibose showed no direct anti-proliferative effect on human CRC cells *in vitro*, underscoring that its antitumor activity is not due to direct cytotoxicity but is mediated through systemic or local microenvironmental changes (Kato et al., 2020).

#### 9.3.2 Key mechanistic pathways

##### 9.3.2.1 Improvement of metabolic parameters and oxidative stress

AGI treatment improves key metabolic parameters and attenuates systemic oxidative stress—two critical drivers of a pro-tumorigenic microenvironment—thereby suppressing colorectal carcinogenesis (Kato et al., 2020).

##### 9.3.2.2 Modulation of local growth factor signaling

A significant downregulation of insulin-like growth factor-1 (IGF-1) mRNA expression was observed in the colonic mucosa of treated mice, suggesting local suppression of a key pathway linking hyperinsulinemia to cancer progression (Kato et al., 2020).

##### 9.3.2.3 Reduction of serum iron levels

Clinical data indicate that AGI use is associated with a significant decrease in serum iron levels in T2DM patients. Elevated iron can induce colonic oxidative stress and epithelial proliferation, thus, iron reduction may represent an additional mechanistic pathway underlying AGI-mediated CRC risk reduction (Xia et al., 2025).

### 9.4 Clinical implications and future perspectives

The following clinical suggestions are based primarily on observational studies from East Asian populations and preclinical mechanistic data. Readers should interpret these recommendations as exploratory, as causality has not been established and findings may not generalize to other populations.

#### 9.4.1 Clinical positioning

For T2DM patients at high risk for CRC (e.g., the elderly, those with a personal history of polyps, strong family history, or hypertension), the choice of an AGI may offer a dual benefit of glycemic control and potential chemoprevention, particularly when used at adequate doses for a sustained period. The strong dose-response relationship suggests that achieving sufficient cumulative exposure is important for any potential protective effect.

#### 9.4.2 Consideration of limitations

While the observational data are promising, several important limitations must be considered when interpreting these findings for clinical practice. Notably, the protective association is not uniform across all studies. Indeed, conflicting evidence exists: a large population-based nested case-control study involving over 2 million T2DM patients found no significant association between AGI use and CRC risk (aOR not reported as significant), contrasting with the consistent protective signals from cohorts (Shin et al., 2020; Tseng et al., 2015; Xia et al., 2025; Horibe et al., 2017). This heterogeneity may be attributed to differences in study population characteristics (e.g., age distribution, comorbidity profiles) or AGI exposure assessment (e.g., cumulative dose vs. binary use). This discrepancy underscores the need for further confirmation.

Furthermore, the data are predominantly derived from observational studies in Asian populations. While consistent and showing dose-response, they cannot definitively establish causality due to potential residual confounding.

AGIs are not first-line therapy for T2DM in most guidelines due to their modest glycemic efficacy and frequent gastrointestinal side effects (e.g., flatulence, diarrhea). Therefore, their potential oncological benefit should be considered a secondary advantage within a comprehensive, individualized diabetes management plan, not a primary indication for prescription.

#### 9.4.3 Future research priorities

1. Conduct prospective cohort studies and RCTs in diverse ethnic populations to confirm the causal relationship.
2. Explore whether the protective effect is analog-specific (e.g., acarbose vs. voglibose).
3. Further elucidate the precise relative contributions of systemic metabolic improvement versus local intraluminal and mucosal effects.
4. Investigate the potential utility of AGIs in non-diabetic populations at high risk for CRC, given their localized mechanism of action.

## 10 Discussion

This review delineates the diverse pharmacological profiles of antidiabetic drugs in regulating CRC risk, highlighting their potential for oncological repurposing. Beyond glycemic control, these agents act on core signaling pathways including AMPK, PI3K/AKT/mTOR, SIRT3, and PPARγ, which directly modulate cancer cell proliferation, apoptosis, immune microenvironment remodeling, and metastatic potential in CRC. Such pharmacological properties provide a strong mechanistic basis for repurposing antidiabetic drugs as chemopreventive or adjuvant anticancer agents. Our comprehensive synthesis across eight drug classes reveals that each class exhibits distinct, mechanism-driven effects on CRC risk and prognosis, rather than a uniform class effect. These findings underscore the importance of integrating pharmacological insights into clinical decision-making for T2DM patients at elevated CRC risk, and support the rational development of drug repurposing strategies for CRC prevention and therapy.

### 10.1 Principal findings and clinical translation: a risk-stratified framework

The most salient finding of this review is that CRC risk should be an explicit consideration in the selection of glucose-lowering therapy for T2DM patients, particularly those at elevated baseline risk. The evidence robustly supports a risk-stratified framework: Metformin and SGLT-2 inhibitors emerge as the most favorable options, with consistent observational signals of lower CRC risk and improved survival in patients with established disease. In contrast, exogenous insulin and most insulin secretagogues (except gliclazide) are associated with a dose-dependent increase in risk, positioning them as less desirable choices for high-risk patients. GLP-1 RAs present a reassuring class-wide safety profile from RCT data, though the risk signal for high-dose semaglutide necessitates vigilance. DPP-4 inhibitors occupy a controversial middle ground, with evidence too contradictory to support their use for chemoprevention. TZDs and AGIs show promising yet geographically variable protective signals but are limited in clinical utility by their side effect profiles and modest glycemic efficacy.

This stratification supports a paradigm shift in T2DM management: optimizing glycemic control must be balanced with long-term oncological risk mitigation, especially given the shared pathophysiology of T2DM and CRC.

To provide a comprehensive, at-a-glance overview of the evidence synthesis for each antidiabetic drug class, Table 1 summarizes key associations with CRC incidence, strength of evidence, impacts on CRC survival, core mechanistic pathways, and tailored recommendations for T2DM patients at high CRC risk. This table integrates findings from epidemiological, clinical, and preclinical studies to support risk-stratified clinical decision-making.

**TABLE 1:** Pharmacological effects, CRC risk association, and clinical recommendations of antidiabetic drugs.

Drug class	Representative agents	Association with CRC incidence	Key evidence strength	Impact on CRC survival/Outcomes	Core mechanisms	Overall recommendation for high CRC risk patients
Biguanides	Metformin	Consistently protective	Strong: Large-sample observational studies; robust preclinical evidence; Mendelian randomization supports biological plausibility; neutral results in bias-adjusted studies; limited RCTs for cancer prevention	Improves overall survival (OS)/disease-free survival (DFS) in T2DM-CRC patients; synergistic with chemo/radiotherapy; reduces cancer-specific mortality	1. AMPK activation inhibits mTOR signaling; 2. MYC suppression downregulates SLC7A5 (tryptophan transporter) to restore CD8^+^ T-cell function; 3. Modulates gut microbiota (↑Akkermansia, ↑short-chain fatty acids); 4. Inhibits NLRP3 inflammasome; 5. Suppresses urea cycle to reduce putrescine generation	First-line preferred option for T2DM patients; long-term regular use recommended; combine with chemo/radiotherapy for established CRC; adhere to standard CRC screening (colonoscopy every 5 years)
SGLT-2 inhibitors	Dapagliflozin, empagliflozin, canagliflozin	Likely protective	Strong but inconsistent: Multi-center large-sample observational studies show consistent survival benefit; preclinical evidence supports direct cytotoxicity; however, Mendelian randomization shows no clear causal association	Significantly improves OS/PFS in T2DM-CRC patients; reduces CRC recurrence risk; broad-spectrum anticancer effects	1. SIRT3-mediated mitochondrial stress induces CRC cell apoptosis; 2. Indirect anti-inflammatory effects (↓TNF-α, ↓macrophage infiltration) and metabolic improvements (↓blood glucose/body weight); 3. Inhibits glucose uptake in CRC cells to block energy supply	Favorable choice, especially vs. DPP-4 inhibitors/insulin secretagogues; suitable for T2DM patients with obesity/family history of CRC; long-term use acceptable if no contraindications; confirm with long-term RCTs
GLP-1 receptor agonists	Liraglutide, dulaglutide, semaglutide	Largely neutral; lower risk compared to DPP-4 inhibitors	Moderate: RCTs show no class-wide increased risk; observational data confounded by weight loss; high certainty of evidence; notable dose-dependent signal for high-dose semaglutide	Limited direct evidence; indirect benefit via weight loss; no consistent OS/DFS improvement in established CRC	1. Species-specific effect: GLP-1R activation induces apoptosis in murine models; 2. GLP-1R often absent in human CRC, limiting direct effects; 3. Indirect effects: weight loss improves metabolic disorders and inhibits intestinal inflammation	Generally safe for use; enhanced CRC screening (consider annual colonoscopy) for patients on high-dose semaglutide; preferred for high-risk patients needing weight reduction; avoid as first-line for CRC chemoprevention
DPP-4 inhibitors	Sitagliptin, linagliptin, Saxagliptin	Controversial (neutral to slightly increased risk); J-shaped dose-response; higher risk in males/<65 years	Moderate and conflicting: RCTs suggest protective effects; observational studies show harm/neutrality; insufficient long-term data; significant study design heterogeneity	Conflicting: worse DFS in some studies (HR=1.98); improved OS when combined with metformin; no consistent benefit in advanced CRC	1. Anti-tumor: Inhibits CXCL5/SDF-1 signaling to reduce invasion; 2. Pro-tumor: Accelerates EMT (↑Zeb1), reduces CD3+/CD8+ T-cell infiltration, increases M2 macrophages; 3. Dose-dependent dual effects	Not a first-line option; avoid long-term high-dose use; combine with metformin if necessary; strict adherence to CRC screening (colonoscopy every 5 years); prioritize SGLT-2 inhibitors/GLP-1 RAs as alternatives
Insulin	Human insulin, insulin glargine	Consistently increased risk; insulin glargine shows clinical neutrality vs. *in vitro* high-dose risk	Strong: Large-sample observational studies; Mendelian randomization confirms causal association; consistent dose-response relationship; conflicting preclinical vs. clinical data for glargine	Worse OS in CRC patients; risk modifiable with metformin combination; increases recurrence risk in advanced CRC	1. Hyperinsulinemia activates IGF-1R/PI3K/AKT/mTOR signaling to promote proliferation; 2. Insulin glargine upregulates oncogenic miR-95 (*in vitro* high dose); 3. Inhibits apoptosis and stimulates angiogenesis	Last-line therapy; co-prescribe metformin (reduces CRC risk by ∼50%) if insulin is unavoidable; enhance CRC screening (initiate at 45 years, colonoscopy every 5 years); avoid long-term high-dose use
Thiazolidinediones	Rosiglitazone, pioglitazone	Modestly protective	Moderate and heterogeneous: Strong observational data from Asian cohorts; preclinical synergy with chemotherapy; high heterogeneity in Western populations; limited clinical outcome data	Preclinical support as chemo-sensitizer; no consistent OS/DFS improvement in clinical studies; pioglitazone associated with bladder cancer risk	1. PPARγ activation inhibits EMT and reduces NF-κB-mediated inflammation; 2. Agent-specific: Rosiglitazone > pioglitazone in protective effects; 3. Suppresses CSC markers (CD133/CD44) and promotes MET	Potential adjuvant therapy in oncology (combined with 5-FU/PD-1 inhibitors); not recommended for sole CRC chemoprevention; rosiglitazone preferred for high-risk Asian T2DM patients; monitor bladder function for pioglitazone users
Insulin secretagogues	Glimepiride, glibenclamide, gliclazide	Class effect: Increased risk; exception: Gliclazide is protective	Moderate and analog-specific: Observational data show high heterogeneity; gliclazide has unique anti-inflammatory mechanism; age-dependent risk	Limited data; gliclazide may benefit inflammation-driven CRC; no survival improvement for other sulfonylureas	1. Gliclazide: Activates AMPK/NF-κB anti-inflammatory pathway, reduces TNF-α, increases IL-10; 2. Other sulfonylureas: Induce hyperinsulinemia via insulin secretion, activating IGF-1R signaling	Avoid most sulfonylureas/glinides; gliclazide is the preferred choice if a secretagogue is needed; enhance CRC screening for elderly users (≥65 years); prioritize metformin/SGLT-2 inhibitors as first-line
Alpha-glucosidase inhibitors	Acarbose, voglibose	Likely protective; consistent in East Asian populations	Moderate: Consistent observational data from East Asian cohorts; strong dose-response relationship; evidence from adenoma prevention studies; limited data in Western populations	Limited direct evidence; primarily protective against precancerous adenomas; no confirmed benefit in advanced CRC	1. Improves metabolic parameters (↓blood glucose) and reduces systemic oxidative stress; 2. Downregulates IGF-1 mRNA expression in colonic mucosa; 3. Reduces serum iron levels to mitigate colonic oxidative stress	Suitable for elderly high-risk Asian patients (hypertensive patients may benefit more); not a first-line option (modest glycemic efficacy, GI side effects); combine with other protective agents; adhere to adenoma screening

It is important to emphasize that the clinical recommendations summarized in Table 1 and discussed throughout Sections 2–9 are based predominantly on observational evidence and mechanistic plausibility. Unless explicitly noted as supported by RCTs (e.g., the safety of GLP-1 RAs for gastrointestinal cancers), these suggestions should be interpreted as exploratory and hypothesis-generating rather than definitive practice guidelines. Clinicians should integrate these considerations with individual patient characteristics and preferences, and await confirmation from ongoing and future randomized controlled trials.

### 10.2 Mechanistic plausibility and the translational divide

The epidemiological associations are largely congruent with established and proposed biological mechanisms, reinforcing biological plausibility for a causal relationship. The pro-carcinogenic effect of insulin and sulfonylureas is strongly underpinned by the hyperinsulinemia-driven activation of the PI3K/AKT/mTOR and MAPK pathways via insulin/IGF-1 receptors. Conversely, the protective mechanisms of metformin are pleiotropic, involving direct AMPK-mediated metabolic reprogramming, immunomodulation of the tumor microenvironment, and gut microbiota alterations, providing a multifaceted basis for its observed benefits.

However, a critical translational divide is evident with GLP-1 RAs. While compelling anti-tumor effects are demonstrated in preclinical models, these are contingent on GLP-1R expression. The frequent absence of functional GLP-1R in human CRC tissue fundamentally explains the lack of a corresponding protective epidemiological signal and argues against a direct receptor-mediated carcinogenic effect. This highlights a crucial principle: anti-tumor activity in rodent models does not automatically translate to cancer risk modulation in humans, and *vice versa*. The effects of SGLT-2 inhibitors may represent a more integrated model, combining direct (e.g., SIRT3-mediated mitochondrial stress) and indirect (systemic metabolic and anti-inflammatory improvement) pathways to explain their beneficial observational profile.

Despite robust preclinical mechanistic evidence, a substantial translational gap exists between experimental models and human clinical outcomes. Many antidiabetic drugs show potent anti-tumor activity in cell lines and mouse models, but these effects are often not replicated in human randomized controlled trials or epidemiological studies. Key reasons for this gap include: suprapharmacological drug doses used in preclinical studies, species-specific differences in receptor expression (e.g., GLP-1R in human CRC), lack of clinically relevant tumor microenvironments *in vitro*, and the inability of short-term experiments to recapitulate the long latency of human colorectal carcinogenesis. These limitations mean that preclinical findings support biological plausibility but do not guarantee clinical efficacy or causality in humans. Conversely, a recent transcriptomics-based study identified eight shared key genes between T2D and CRC (including CD44, PTK2, and THBS1) and proposed that certain anticancer agents may have therapeutic potential for both diseases, highlighting a bidirectional drug repurposing perspective (Ahmmed et al., 2026).

**FIGURE 1:**
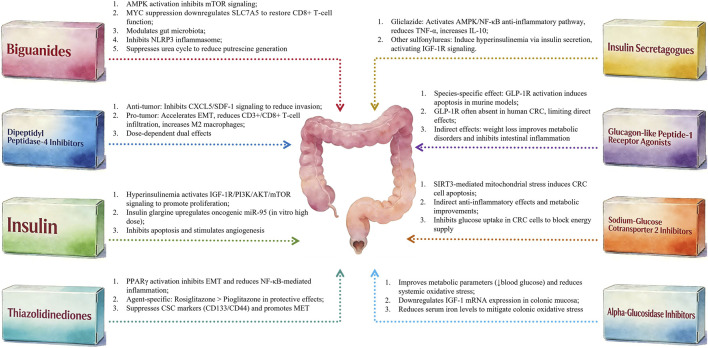
Core mechanisms of major antidiabetic drug classes in regulating CRC risk.

Figure 1 illustrates the key mechanistic pathways through which antidiabetic drug classes modulate CRC risk, highlighting both pro-tumorigenic and anti-tumorigenic effects. This visual summary aligns with the narrative mechanistic discussion, emphasizing conserved pathways (e.g., PI3K/AKT/mTOR activation by insulin) and drug-specific differences (e.g., AMPK/NF-κB inhibition by gliclazide vs. other insulin secretagogues).

### 10.3 Reconciling contradictions: the case of DPP-4 inhibitors and study design

The most striking contradiction in the evidence surrounds DPP-4 inhibitors, where meta-analyses of RCTs suggest protection while observational studies indicate neutral or increased risk. This conflict is likely not biological but methodological. RCTs, the gold standard for efficacy, are typically short-term (2–5 years), underpowered for cancer endpoints, and enroll selected populations, potentially missing long-term or subgroup-specific effects. Observational studies, capable of assessing long-term real-world use, are severely confounded by “confounding by indication.” DPP-4 inhibitors are often prescribed to older, frailer patients with longer diabetes duration and more comorbidities—a profile inherently associated with higher cancer risk and mortality. This residual confounding can create a spurious signal of harm. The duality of DPP-4’s biological function (cleaving both incretins and immunomodulatory chemokines) provides a plausible basis for either net benefit or harm, depending on the patient’s immune context. Therefore, the current evidence does not resolve the risk but underscores the insufficiency of both study designs alone to answer this question.

Geographic heterogeneity, particularly for TZDs and AGIs, further complicates the picture. The stronger protective signals in Asian versus Western cohorts may reflect differences in genetic polymorphisms (e.g., in PPARγ), dietary patterns, body composition, or local prescribing practices and comparator drugs. This heterogeneity mandates that global treatment guidelines incorporate locally relevant evidence.

This methodological dichotomy—RCTs underpowered for cancer outcomes versus observational studies confounded by indication—is not unique to DPP-4 inhibitors. For metformin, a similar pattern emerges: conventional observational analyses report strong protective associations, while bias-adjusted studies (target trial emulation, time-dependent Cox) show neutral effects (Dickerman et al., 2023; Zhang et al., 2022). The examples of SGLT-2 inhibitors and GLP-1 receptor agonists further illustrate this recurring theme. For SGLT-2 inhibitors, the discrepancy between observational associations and Mendelian randomization null findings highlights the complementary limitations of each approach—observational studies capture real-world drug use but are prone to confounding, whereas Mendelian randomization avoids confounding but relies on genetic proxies that may not fully represent pharmacological effects. For GLP-1 RAs, the variability across observational studies underscores the critical roles of comparator choice, weight loss confounding, and detection bias.

### 10.4 Strengths, limitations, and the imperative for better evidence

This review’s strength lies in its comprehensive, cross-class comparison and integration of epidemiological, clinical, and mechanistic data. However, it also reveals the profound limitations of the extant evidence base that constrain definitive conclusions.

The predominance of observational data is the foremost limitation. Despite sophisticated statistical adjustments, residual confounding by unmeasured or imperfectly measured factors (e.g., diet, physical activity, socioeconomic status, diabetes severity) remains a persistent challenge to validity.

To aid readers in interpreting the findings presented throughout this review, it is essential to explicitly outline how specific limitations of observational studies affect the validity of reported associations.

First, immortal time bias occurs when the time period between cohort entry (e.g., diabetes diagnosis) and drug initiation is misclassified as exposed time. This bias systematically overestimates protective effects, as demonstrated by metformin studies where conventional analyses reported strong risk reductions (RR = 0.62–0.88), whereas bias-adjusted methods (time-dependent Cox regression, target trial emulation) showed neutral effects (Dickerman et al., 2023; Zhang et al., 2022).

Second, confounding by indication arises when the indication for prescribing a drug is itself associated with the outcome. For example, metformin is preferentially prescribed to younger, healthier, and more adherent patients, whereas insulin and sulfonylureas are often used in patients with longer diabetes duration and more comorbidities. This can create a spurious protective signal for metformin and an artificially elevated risk for insulin and secretagogues. Readers should recognize that some of the observed ‘benefits’ or ‘harms’ may reflect baseline patient characteristics rather than true drug effects.

Third, residual confounding persists even after statistical adjustment for measured covariates. Unmeasured or imperfectly measured factors—such as diet, physical activity, body mass index, smoking, medication adherence, and socioeconomic status—may confound observed associations. The direction of residual confounding is unpredictable but often biases results away from the null, exaggerating both protective and harmful associations.

Fourth, detection bias is particularly relevant for drugs with gastrointestinal side effects. Glucagon-like peptide-1 receptor agonists, for instance, frequently cause nausea and constipation, prompting increased colonoscopy utilization. This leads to higher detection rates of pre-existing, asymptomatic colorectal neoplasms, creating a spurious signal of increased CRC risk that is not attributable to the drug itself (Silverii et al., 2025).

In summary, while observational studies provide valuable real-world evidence, their limitations—immortal time bias, confounding by indication, residual confounding, and detection bias—can substantially distort effect estimates. Readers should interpret the associations reported in this review with these methodological caveats in mind, and recognize that definitive causal inference requires confirmation from large-scale, long-term randomized controlled trials.

Furthermore, significant heterogeneity across populations, drug analogs, exposure definitions, and follow-up duration obscures the true effect size (Hajishah et al., 2025). The near-total absence of long-term, prospective RCTs with CRC as a primary endpoint represents the fundamental evidence gap. Existing diabetes or cardiovascular outcome trials are neither designed nor powered to address cancer risk reliably.

To address the identified limitations and advance the field, Table 2 systematically outlines major evidence gaps for each antidiabetic drug class and corresponding priority research directions. These directions prioritize definitive clinical trials, mechanistic validation, and personalized medicine approaches to resolve uncertainties and improve translational impact.

**TABLE 2:** Key evidence gaps and proposed future research directions.

Drug class	Major current limitations	Priority research directions
Biguanides	• Observational bias (immortal time, confounding)	1. Large-scale RCTs for CRC prevention and treatment
	• Inconsistent clinical trial results	2. Biomarker-driven patient stratification (e.g., VFI, genetic subtypes)
	• Non-physiological doses in preclinical studies	3. Synergy trials with immunotherapy/targeted therapy
SGLT-2 inhibitors	• Lack of RCTs with cancer endpoints	1. Prospective RCTs with CRC incidence as endpoint
	• Heterogeneity across studies	2. Mechanistic studies on SGLT2/SIRT3/DPP4 axis
	• Unclear direct vs. indirect mechanism contribution	3. Combination therapy trials (with chemo/immunotherapy)
GLP-1 receptor agonists	• Translational gap (GLP-1R often absent in human CRC)	1. Long-term post-marketing surveillance (esp. high-dose semaglutide)
	• Confounding by weight loss and detection bias	2. Studies dissecting drug vs. weight loss effects
	• Dose-dependent safety signal for high-dose semaglutide	3. Exploration of GLP-1R-independent mechanisms
DPP-4 inhibitors	• Stark RCT vs. observational discrepancy	1. Long-term prospective cohorts with biomarker integration
	• Confounding by indication (older, frailer patients)	2. Personalized medicine: tumor CD26/immune profiling
	• Clinical implications of J-shaped dose-response unclear	3. Mechanism-validation studies in clinical cohorts
Insulin	• Formulation-specific risk unclear (glargine controversy)	1. Prospective studies with detailed insulin regimen data
	• Difficult to disentangle risk from T2DM severity	2. Physiologically relevant *in vivo* mechanistic studies
	• Discrepancy between in vitro (high-dose) and clinical data for glargine	3. Trials of insulin-sparing or combination regimens
TZDs	• Geographic heterogeneity (Asia vs. West)	1. Large RCTs for rosiglitazone in CRC prevention/adjuvant therapy
	• Agent-specific differences (rosi- vs. pioglitazone)	2. Biomarker development for patient selection
	• Preclinical pro-tumorigenic paradox	3. Studies on PPARγ context-dependent signaling
Insulin secretagogues	• Analog-specific heterogeneity underappreciated	1. Prospective studies on gliclazide and CRC outcomes
	• Lack of long-term prognosis data	2. Mechanistic dissection of gliclazide vs. other SUs
	• Mechanism for meglitinides unclear	3. Exploration of gliclazide in colitis-associated CRC
AGIs	• Evidence limited to Asian observational studies	1. RCTs in diverse populations for CRC chemoprevention
	• Not first-line therapy (modest efficacy, GI side effects)	2. Analog-specific comparison (acarbose vs. voglibose)
	• Mechanism primarily indirect	3. Exploration in high-risk non-diabetic populations

### 10.5 Implications for clinical practice and future research

#### 10.5.1 Clinical practice

##### 10.5.1.1 Informed decision-making

Clinicians should incorporate individual CRC risk (family history, personal history of polyps, obesity, smoking) into clinical decision-making when selecting antidiabetic therapy. For high-risk patients, metformin or an SGLT-2 inhibitor should be prioritized.

##### 10.5.1.2 Sequencing and combination therapy

When insulin therapy becomes necessary, concerted efforts should be made to co-prescribe metformin, not only for glycemic synergy but also to potentially attenuate the associated cancer risk (Currie et al., 2009).

##### 10.5.1.3 Vigilance, not contraindication

The signals for high-dose semaglutide and DPP-4 inhibitors do not mandate avoidance but should reinforce strict adherence to age-appropriate CRC screening protocols.

#### 10.5.2 Future research priorities

##### 10.5.2.1 Definitive RCTs

There is an urgent need for large, long-term (>10 years) RCTs designed specifically to evaluate CRC incidence as a primary outcome, comparing drugs with divergent risk profiles (e.g., SGLT-2i vs. DPP-4i; metformin vs. sulfonylurea).

##### 10.5.2.2 Precision medicine approaches

Research should move beyond class effects. Studies should identify predictive biomarkers (e.g., tumor GLP-1R status, CD26 expression, visceral fat index, microbiome signatures) to determine which patients will benefit or be harmed by a specific drug.

##### 10.5.2.3 Mechanistic elucidation

For SGLT-2 inhibitors, studies should quantify the relative contribution of direct tumor cell cytotoxicity versus systemic metabolic improvement. For GLP-1 RAs, research should focus on GLP-1R-independent effects.

##### 10.5.2.4 Leveraging real-world data

Advanced analytics applied to large, linked electronic health records and biobanks can provide insights into long-term effects, drug-drug interactions, and analog-specific differences while attempting to better control for confounding.

## 11 Conclusion

T2DM management is evolving from a singular focus on glycemic control to a holistic strategy encompassing the prevention of associated complications, including cancer. This review establishes that antidiabetic medications are not metabolically inert with respect to CRC risk; these agents are active modifiers with divergent risk profiles. While metformin and SGLT-2 inhibitors currently offer the most favorable risk-benefit balance, the field is hampered by a reliance on observational evidence with inherent limitations. Bridging this gap requires a concerted translational effort—encompassing robust clinical trials, mechanistic discovery, and the implementation of personalized medicine—to ensure that T2DM treatment effectively mitigates the burden of CRC.
